# From Wound Dressing to Tissue Regeneration: Bilayer Medicated Patches for Personalized Treatments of Chronic Wounds

**DOI:** 10.1021/acsami.5c06444

**Published:** 2025-06-05

**Authors:** Sara Bernardoni, Elisabetta Campodoni, Gaia Vicinelli, Mohamed Saqawa, Francesca Bonvicini, Laura Pulze, Nicoló Baranzini, Giorgia Costantini, Monica Montesi, Giovanna Angela Gentilomi, Annalisa Grimaldi, Monica Sandri

**Affiliations:** † Institute of Science Technology and Sustainability for Ceramics (ISSMC), 9327 National Research Council (CNR), Via Granarolo 64, Faenza 48018, Italy; ‡ Department of Chemical, Biological, Pharmaceutical and Environmental Sciences, University of Studies of Messina, Messina, ME 98122, Italy; § Department of Pharmacy and Biotechnology, 9296 Alma Mater Studiorum - University of Bologna, Via Massarenti 9, Bologna 40138, Italy; ∥ Department of Biotechnology and Life Science, 19045 University of Insubria, Via Dunant 3, Varese 21100, Italy; ⊥ Microbiology Unit, IRCCS Azienda Ospedaliero-Universitaria di Bologna, Via Massarenti 9, Bologna 40138, Italy

**Keywords:** wound-healing, magnesium-doped hydroxyapatite, tissue regeneration, antimicrobial biomaterials, infection diseases

## Abstract

Chronic wounds pose a significant healthcare challenge, impairing the quality of life for millions of affected individuals. This phenomenon escalates due to the aging of the population and rising comorbidities. Traditional wound care methods often prove inadequate in dealing with the complexities of chronic wounds; therefore, biomaterials have emerged as promising solutions. In response to this need, this work focuses on the development of a bilayered hybrid patch for the treatment of chronic wounds, designed with a chemical composition and morphology to exert antimicrobial activity to combat local infection and to provide specific support for cell adhesion and tissue regeneration. In particular, using gelatin and chitosan as the main constituent materials, bioactive membranes were developed and functionalized with bioresorbable hydroxyapatite nanoparticles doped with magnesium ions grown on gelatin molecules to boost regenerative stimuli. Then, they were assembled into a bilayered structure with highly tuned chemical and structural features through different fabrication techniques and biodegradation by cross-linking processes. Lastly, to confer antibacterial properties, the lower layer was medicated in situ with Vancomycin hydrochloride (VNC), selected as a case study antibiotic. The developed patches exhibit excellent physiochemical properties, including exudate absorption and moisture permeability, with both features falling within the recommended range for materials for wound healing applications. In addition, both patches exhibit adequate biodegradation times to support effective cell adhesion and proliferation, as well as drug release kinetics, with almost complete release of VNC after 48 h, necessary to achieve thorough wound disinfection. In vitro biological studies have proved their biocompatibility and on-site, long-lasting antimicrobial potential, while in vivo tests, with medicinal leeches’ model, have demonstrated their affinity for live tissue and efficacy in supporting endothelial cell proliferation by stimulating the epidermal tissue healing process.

## Graphical abstract

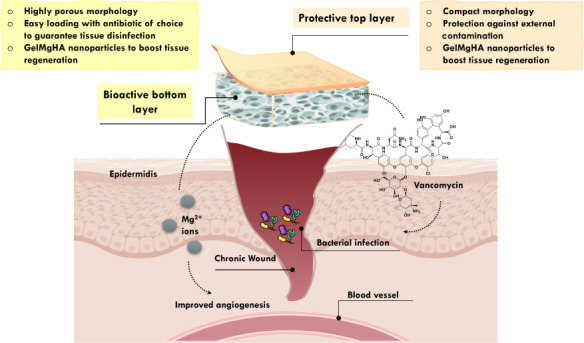

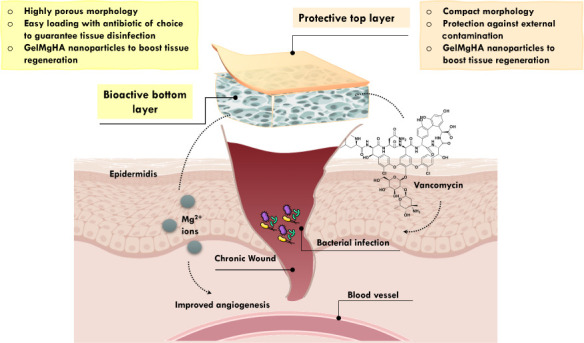

## Introduction

Skin wound injuries are the most common form of trauma; they can stem from a wide range of sources, including damages, pressure, burns, cuts, surgeries, and injuries resulting from underlying pathological conditions, hence the importance of research into innovative treatments. This urgency is even more relevant when associated with pathological conditions like cardiovascular, cerebrovascular, and senile diseases, diabetes, hypoxia, and cancer, which delay the body’s natural repair mechanisms, leading to the insurgence of chronic wounds.^1^ As a result of the prolonged healing process, this kind of lesion is prone to bacterial infection and biofilm formation, triggering sustained abnormal inflammation. Left unaddressed, this scenario can culminate in amputation or even death.^2^ Ineffective treatments resulted, only in Europe, in more than 8 million patients suffering from diabetic foot ulcers each year, with a 5-year mortality rate comparable to that of cancer (31%) and a cost of EUR 2–2.5 billion, underlining the impact on patients’ quality of life and substantial economic loss of the healthcare system.^3^ Therefore, it is crucial to dispose of advanced dressing ensuring the conditions for rapid wound healing, reducing the risk of contamination, and favoring an optimal skin regeneration.^4^ Conventional treatments for chronic wounds include systemic and topical administration of antimicrobial agents, frequent dressing changes, operative debridement, and flaps repair.^5^ However, they come with inherent drawbacks, including the need for anesthesia, pain for patients, and side effects associated with antibiotics administration.^6^ Of particular concern is the growing antibiotic-resistance, which leads to their administration being reserved only for cases justified by comorbidities and critical clinical conditions.^7^

Given the limitations of traditional treatments and the imperative to explore alternative infection control strategies, significant attention has been turned toward biomaterials. Particularly, biomaterials derived from natural sources have garnered considerable interest due to their inherent properties, such as biodegradability, biocompatibility, and bioactivity. Multiple studies have affirmed the antibacterial, eukaryotic cell-proliferative, and immunomodulatory attributes of biobased materials.^2,8^ Further, their ability to be blended and formed into injectable hydrogels and aerogels offers the potential to develop tailor-made devices with enhanced cell adhesion and proliferation properties, appropriate mechanical strength to support the tissue during the healing process, and porous structures that promote vascularization and fluid exchange with the wound site.^4^ Multilayer dressings are receiving increasing attention as they can incorporate the benefits of different morphologies and functions, stimulating a better healing process.^9^ They can be obtained through the combination of different fabrication techniques, including freeze-drying, solvent casting,^10^ electrospinning,^11^ or 3D printing,^12^ as well as the combination of different materials.^13^ This translates into the possibility of developing customized dressings to meet specific wound care needs, making them more effective than single-layer dressings.

Among biopolymers, gelatin, whose molecular structure remarkably mirrors the extracellular matrix, emerges as an elective biomaterial for biomedical and pharmaceutical applications.^14,15^ However, gelatin lacks an inherent antibacterial efficacy to prevent wound infections. To overcome this limitation, it is often combined with other biopolymers to produce a hybrid hydrogel with superior antibacterial effects. Among them, chitosan, a polysaccharide derived from chitin, as it possesses inherent antimicrobial properties, is widely used in biomedical settings and holds particular significance as a wound dressing material due to its documented prowess in promoting wound healing.^14,16^ In recent years, many dressing materials based on gelatin and chitosan have been developed,^17–19^ either alone or in combination with other biopolymers, to address the challenges of chronic wound healing, especially considering the possibility of loading these matrices with a wide variety of therapeutic molecules strategic for accelerating the regenerative process. These therapeutic agents, encompassing antibiotics,^20^ peptides,^21,22^ vitamins,^23^ growth factors,^24^ and antioxidants,^25^ can be delivered directly into the wound via the bioactive dressing. The local delivery offered by biomaterials for wound dressing is crucial as it avoids the difficulties associated with both systemic administration of drugs, which include reduced bioavailability, systemic toxicity, and general side effects, especially in patients with a complex clinical picture, and the use of topical formulations, which are often ineffective due to excessive secretion of exudates and bacterial biofilm.^26^ Indeed, the use of absorbent dressings or biomaterial hydrogels loaded with the drugs of choice has been shown to increase their efficacy by reducing the diluting effect of exudate and physically shielding the drugs from the harsh environment of infected chronic wounds, thereby improving their bioavailability.^27^ Furthermore, they are able to enhance the process of wound healing by actively stimulating tissue regeneration, fostering angiogenesis, and collagen synthesis, while controlling inflammation and preventing infections.^28^ Alongside the delivery of traditional antibiotics, bioactive mineral particles and metal ions present themselves as a valuable alternative to be used in combination with antibiotics to prolong and enhance the dressing’s antibacterial activity and healing potential.^26,29^

Among mineral particles, hydroxyapatite, the predominant component in hard tissues, for its high biocompatibility, bioactivity, and biodegradability, is considered a preferred biomaterial to confer multiple functionalities to composite biomaterial formulations, especially for bone tissue regeneration.^30^ Its ability to incorporate foreign ions within its crystalline structure offers the possibility to significantly enhance its bioactivity, expanding its potential applications, encompass the treatment of infections and wounds.^31^

With this study, our purpose was to design and validate an innovative multilayer dressing for the treatment of difficult-to-heal wounds, capable of recreating an ideal microenvironment to promote the processes of disinfection and regeneration of dermal tissue. It was obtained by layering a compact outer layer, which mimics the epidermis and provides a protective barrier against external contaminants while ensuring the vapor permeability required for wound dressing materials, with a porous inner layer, similar to dermal tissue. The latter is bioresorbable and, being highly porous, capable of absorbing wound exudates, ensuring cell permeation and proliferation, and allowing the loading and release of therapeutic agents for on-site antimicrobial wound disinfection. In addition, to provide the inner layer with long-term regenerative stimuli, it was supplemented with nanostructured mineral particles as a reservoir of magnesium ions, whose concentration is crucial in soft tissue treatment, as it influences the migration and adhesion of human skin fibroblasts (HSFs) and promotes angiogenesis, a vital process in wound healing.^32,33^ For this purpose, bioactive particles were specifically synthesized through a synthesis process that mimics nature-inspired biomineralization and enables the growth of biomimetic hydroxyapatite doped with magnesium ions (Mg^2+^) on gelatin molecules (GelMgHA), resulting in a bioresorbable nanostructured mineral phase that closely mimics biological apatite.^34^ Its ability to degrade in a physiological microenvironment enables the sustained release of Mg^2+^ ions hosted in the HA lattice.

The two distinct layers were developed by incorporating GelMgHA hybrid particles into two formulations of polymer blends: one of gelatin and chitosan, and one of chitosan and glycerol, the latter as a plasticizer for an easier adhesion to the wound site, and finally assembled as 3D multilayer patches with finely tuned chemical and structural features. The structure of the layers was modulated through different fabrication techniques (freeze-drying and solvent casting), and their degradation kinetics were controlled through cross-linking procedures. Each individual layer underwent comprehensive characterization, which assessed its suitability as a wound dressing material by evaluating parameters such as fluid handling capacity, degradation time, water-vapor transmission rate, and porosity, and was then integrated into a bilayer structure designed to mimic the natural structure and function of skin tissue.^35^

In addition, we set up a protocol to medicate the patches with an antibiotic drug to actively fight infections in chronic wounds; Vancomycin hydrochloride (VNC), a common antibiotic effective against S. aureus and coagulase-negative staphylococci,^36^ was selected for the study. The loading process has been developed to simulate a possible procedure in a medical setting immediately prior to the application of the dressing, ensuring a personalized therapeutic strategy tailored to the patient’s need and on the microorganisms responsible for the infection.^37^

This design approach is thought to merge the local delivery of antibiotics, strategically loaded into the device, with the prolonged release of bioactive Mg^2+^ ions to pursue the tissue regeneration processes. To demonstrate the biocompatibility, antimicrobial activity, and efficacy of dressing devices in promoting cell adhesion and proliferation, and stimulating epidermal tissue regeneration, an in vitro test, with human skin fibroblasts and S. aureus and S. epidermidis, and in vivo investigations with medicinal leeches were performed.

## Results and Discussion

With the recent surge of interest in biomaterials’ design for skin-restoring purposes, several efforts have been made to design biomaterials that support tissue regeneration by providing conducive microenvironments for cell adhesion, proliferation, and differentiation. These materials, which can be functionalized with active agents like antibiotics, antimicrobial peptides, or bioactive ions to act for targeted drug delivery, have gathered considerable attention, particularly in the domain of chronic wound healing, where sustained and localized therapeutic application stands as a preferred approach over systemic administration.^38^

Particularly, the opportunity of easily loading the material directly in a clinical setting, before the dressing application, poses an interesting prospect, as it allows tailoring of the drug selection based on the necessity of the patient, moving toward the concept of personalized medicine.

In this context, our study aims to create a bioresorbable 3D matrix that is able to provide simultaneous wound disinfection and healing. This is achieved through the loading and targeted administration of an antibiotic, such as Vancomycin, while concurrently exploiting the ability of the material to act as a biomimetic and bioactive support for tissue regeneration. For this issue, hybrid nanoparticles of hydroxyapatite doped with magnesium ions to boost regeneration and angiogenesis were synthesized and used as bioactive additives in a biopolymer hydrogel mainly composed of chitosan, gelatin, and glycerol as plasticizer.

To comprehensively explore the influence of polymer composition and material morphology, three distinct single-layered patches were developed utilizing two different polymeric hydrogels composed of gelatin and chitosan in a 3:1 weight ratio, and of chitosan and glycerol in a 2:1 weight ratio. Additionally, the samples were fabricated through two distinct manufacturing methods, freeze-drying and solvent casting, which allowed for precise modulation of the porosity of the patch, resulting in highly porous samples in one case and compact membranes in the other. The diverse morphologies obtained enabled the assessment of the porosity contribution. Moreover, these single-layer patches were thoroughly evaluated in terms of their ability to act as an effective local drug delivery system for the treatment of chronic wounds.

### Development and Characterization of GelMgHA Hybrid Particles

Adopting a biomimetic approach, a biomineralization process was carried out on gelatin (Gel) molecules to synthesize biohybrid particles in which both the mineral and the organic phases mimic the characteristics of the natural apatite. During the synthesis process, Ca^2+^ ions interact with the carboxylic functional groups of Gel, initiating the nucleation of apatite nanocrystals on the polymeric matrix (Figure 1). This reaction, in the presence of Mg^2+^ ions, facilitates the nucleation of an almost amorphous Mg-doped nanostructured hydroxyapatite (MgHA), faithfully replicating both the chemical and the physical attributes of natural apatite, thus generating mineralized hybrid particles where apatite is highly bioresorbable due to its low crystallinity.^34,39^

**Figure 1:**
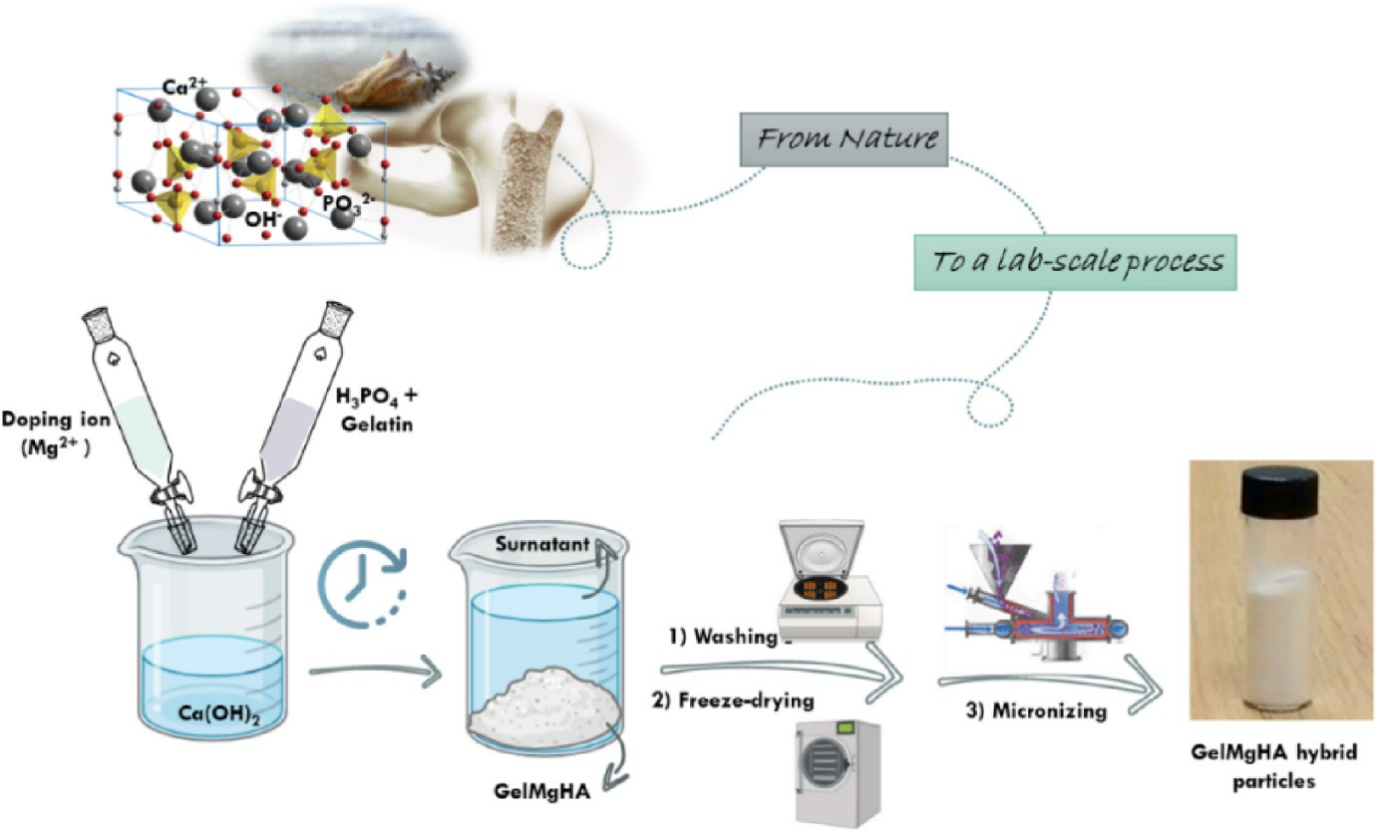
Schematic representation of the nature-inspired biomineralization process for the development of GelMgHA hybrid particles.

FEG-SEM image (Figure 2a) shows the presence of needle-like aggregates of the mineral phase enveloping the Gel structure, forming flakes of about 1 μm. ICP analyses (Figure 2b) confirm the presence and quantify the amount of Mg^2+^ ions within the hybrid particles at a molar ratio of Mg/Ca = 0.06, essential to enhance the biomimicry of natural tissue and favor the angiogenetic processes. The molar ratio (Mg + Ca)/P is 1.63, while Ca/P ratio is 1.54, indicating a deviation from the theoretical ratio of the crystalline apatite (Ca/P = 1.67). This supports both the partial substitution of Ca^2+^ with Mg^2+^ and the mineralization of a low-crystalline apatite onto the gelatin, likely due to its interaction with the organic template. This is further confirmed by XRD spectra (Figure 2c), revealing a broadened diffraction pattern typical of low-crystalline apatite phases, in contrast to the significantly more resolved diffraction spectrum reported and obtained for MgHA prepared under the same conditions but without the Gel.

**Figure 2:**
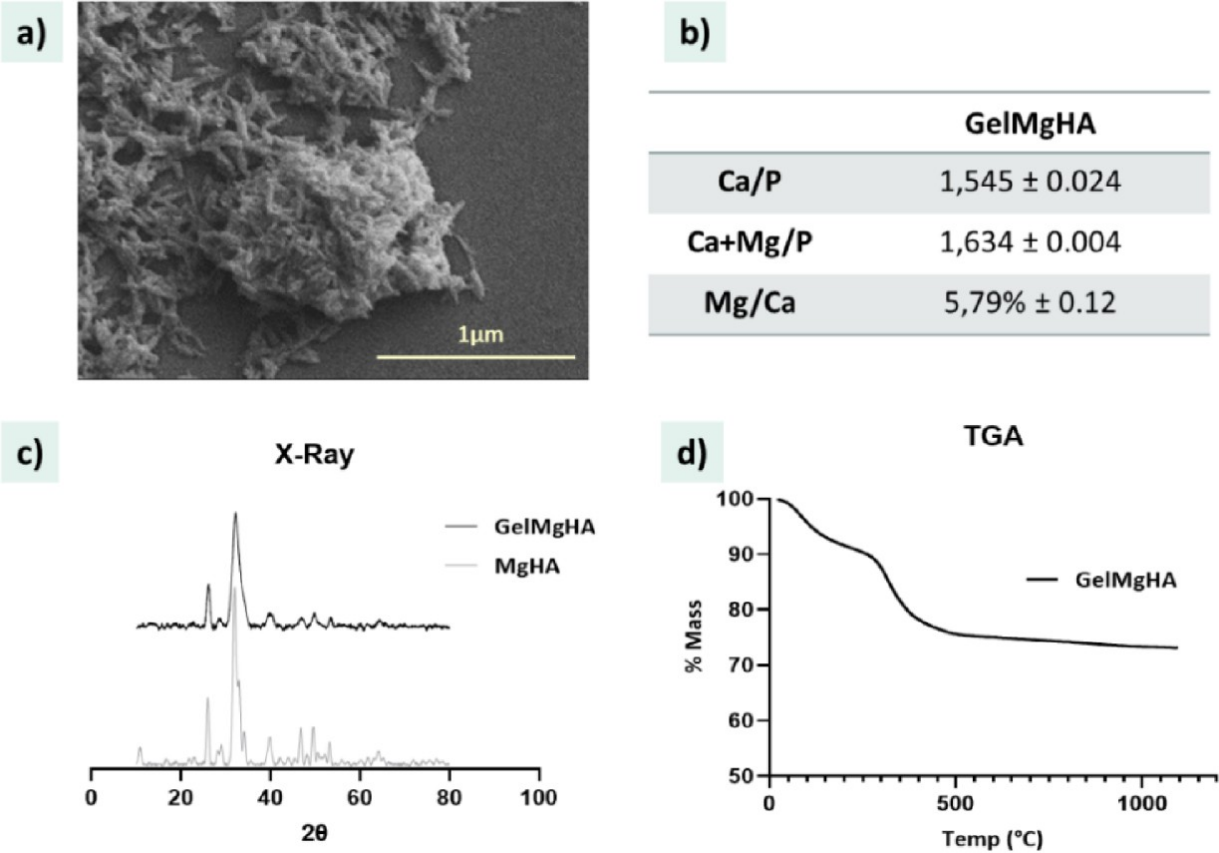
Morphological and chemical characterizations of GelMgHA hybrid particles. a) FEG-SEM analyses of the morphology of the hybrid GelMgHA agglomerated particles; b) chemical composition analyzed with ICP; c) X-ray diffraction patterns of GelMgHA compared to MgHA as control; d) thermal degradation curve obtained by TGA.

TGA analysis confirmed the content of about 15% of Gel inside the clustered particles and 80% of MgHA (Figure 2d) essential to achieve an adequate amount of mineral phase in the 3D membranes obtained by mixing those particles with the polymeric matrix.

### Development and Characterization of Hybrid Single-Layer Patches

The biomimetic GelMgHA particles have been involved in the development of three different hybrid, single-layer patches, varying in composition, morphology, and microstructure (GelMgHA@GelChit3:1_freeze, GelMgHA@ChitGly2:1_freeze, GelMgHA@ChitGly2:1_casting). They are embedded into two different compositions of polymeric hydrogels, developed by selecting chitosan, gelatin, and glycerol as biocompatible polymers, to achieve a GelMgHA/polymer ratio of 30/70, as reported in the Table 5 of the Materials and Methods section. Then, freeze-drying and solvent casting processes were used to fabricate bilayered 3D patches (scaffolds) with different morphology, microstructure, and behavior.

#### Morphological Characterization

The biomaterial porosity and microstructure play a crucial role in regenerative medicine, allowing cell adhesion, penetration, proliferation, and permeation of nutrients and oxygen. As resulted by electron microscopy morphological analysis of longitudinal sections (Figure 3), a highly porous structure with an open and interconnected network was observed for the patches prepared through freeze-drying (GelMgHA@GelChit3:1_freeze and GelMgHA@ChitGly2:1_freeze), characterized from a pore size of 131.7 ± 38.8 μm (Figure 3a, GelMgHA@GelChit3:1_freeze) and of 105.0 ± 35.3 μm (Figure 3b, GelMgHA@ChitGly2:1_freeze). On the contrary, the patch obtained through solvent casting techniques (Figure 3c, GelMgHA@ChitGly2:1_casting) exhibited a smooth and compact surface with no evident porosity inside the scaffold. These findings align with the macroporosity assessment conducted using the water-squeezing method. Macroporosity holds significant importance, as it represents the space available for effective cell penetration and migration within the scaffold. Both freeze-dried patches exhibit high macroporosity values exceeding 80%. GelMgHA@ChitGly2:1_freeze demonstrates the highest value at 87.0 ± 0.4%, while GelMgHA@GelChit3:1_freeze exhibits a slightly lower macroporosity of 81.6 ± 0.6%. Conversely, the air-dried patch GelMgHA@GelChit3:1_casting, due to its smooth surface and compact structure, shows significantly lower macroporosity, measuring at 36.5 ± 6.8%.

**Figure 3:**
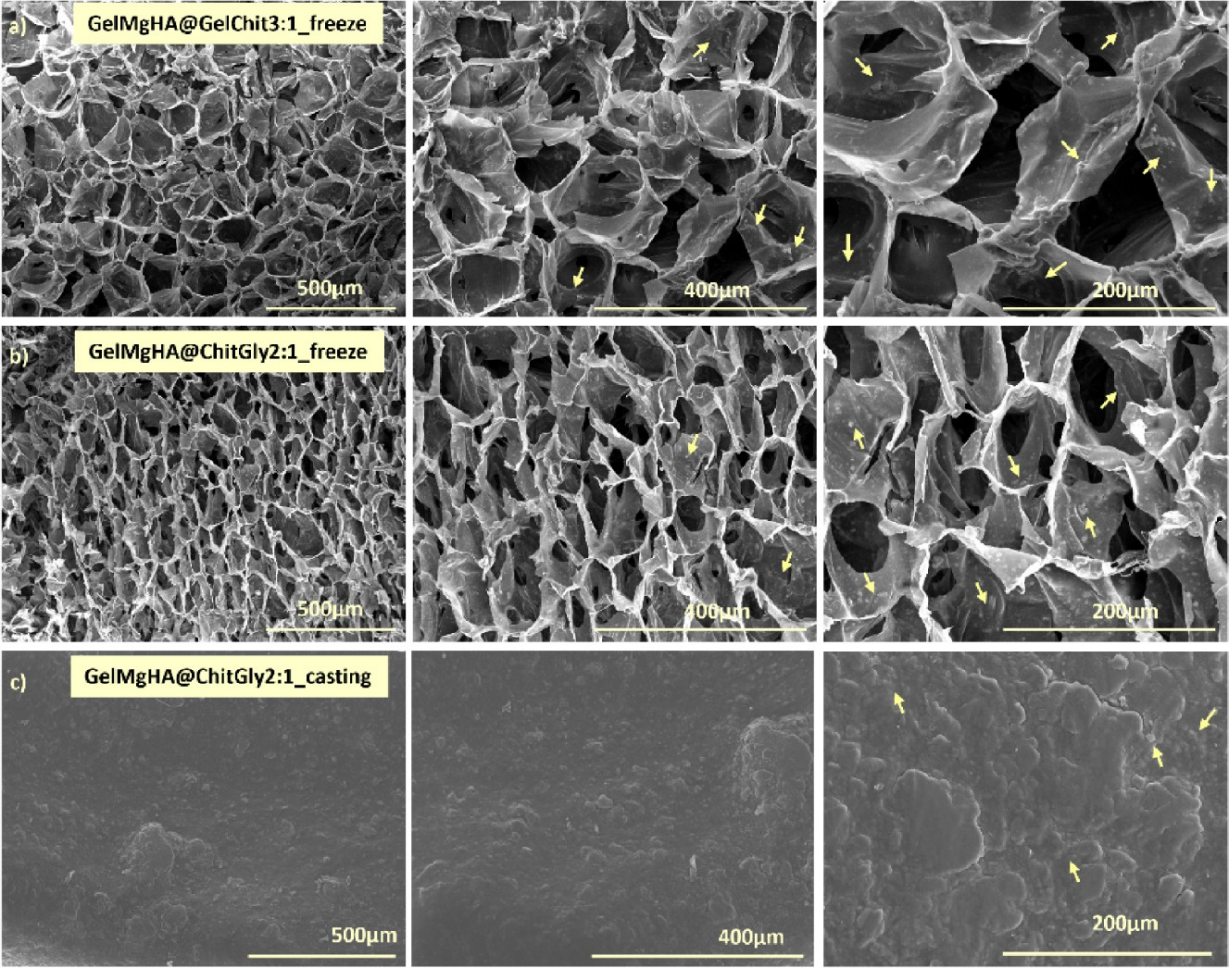
Morphological evaluation of hybrid single-layer patches. Different magnifications are reported in the panel to better appreciate the whole morphology of the patches. a) GelMgHA@GelChit3:1_freeze, b) GelMgHA@ChitGly2:1_freeze, and c) GelMgHA@ChitGly2:1_casting. Yellow arrows indicate the GelMgHA hybrid particles.

ESEM morphological analysis at higher magnification (Figure 3, right column) highlights that the GelMgHA particles are well-integrated and uniformly distributed in both polymeric matrices and are clearly visible in the roughness on the patch surface or on the wall of the pores.

#### Chemical Characterization

Despite the blending of the hybrid particles GelMgHA into the polymeric hydrogels, GelMgHA@GelChit3:1_freeze, GelMgHA@ChitGly2:1_freeze, and GelMgHA@ChitGly2:1_casting, the mineral phase maintains intact its chemical properties. As ICP revealed (Figure 4a), the final patches maintained a Ca/P mole ratio of 1.58, similar to that found for GelMgHA particles and typical of low-crystalline hydroxyapatite partially substituted with Mg^2+^ (Mg/Ca about 5.7%). Moreover, XRD analysis (Figure 4b) affirmed the retention of low-crystalline MgHA, exhibiting the characteristic pattern marked by broad peaks.

**Figure 4:**
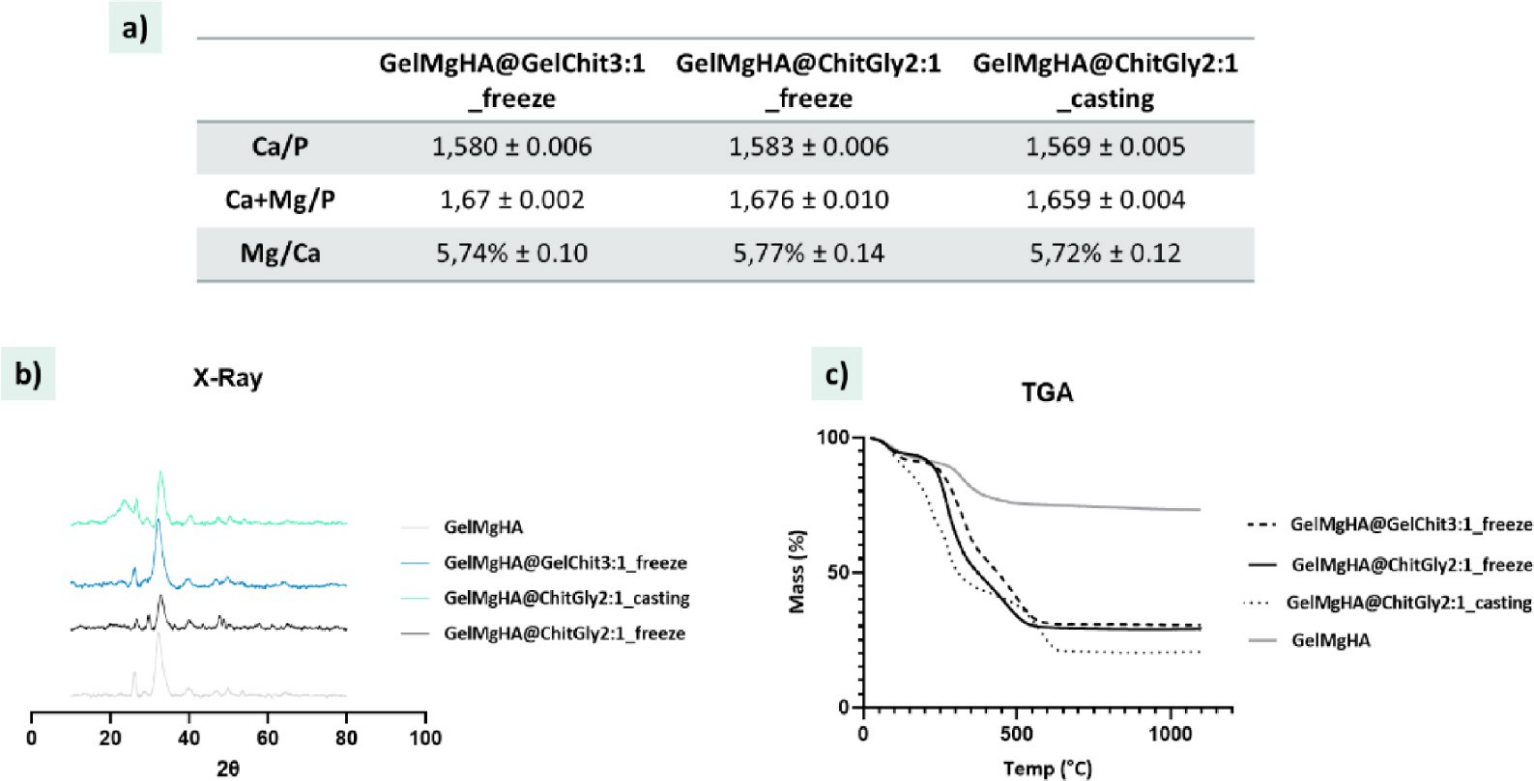
Chemical evaluation of the hybrid single-layer patches. a) Chemical composition obtained from ICP analysis; b) X-ray diffraction patterns of the developed single-layer patches compared to GelMgHA hybrid particles as control; c) single-layer patches thermal degradation curves obtained by TGA, plotted against the thermal degradation curve of GelMgHA hybrid particles as control.

Thermogravimetric analysis revealed an overall content of MgHA mineral phase of 30 wt % in the freeze-dried patches (Figure 4c). On the other hand, the air-dried patch containing chitosan and glycerol (GelMgHA@ChitGly2:1_casting) shows weight losses such that the residual mineral phase content appears to be 20 wt %. However, since they were obtained from the same blend, this can be ascribed to a partial loss of the glycerol during the freeze-drying and cross-linking process, which does not happen during air-drying. In fact, for the patches containing glycerol, the amount of mineral phase to be incorporated was estimated in relation to the amount of chitosan alone and does not factor into the added glycerol as a plasticizer.

The TGA profiles reveal distinctive weight losses, ascribable to the breakdown of inter- and intramolecular hydrogen bonds between polymers and to water release. In particular, up to 150 °C, the weight loss is attributed to the evaporation of residual water molecules; between 200 and 300 °C, the degradation of glycerol occurs, while the final weight loss between 300 and 600 °C is ascribed to the breakdown of the polymeric components (gelatin and chitosan).

#### Physical Evaluation

To support optimal wound healing, biomaterials must effectively regulate moisture by either retaining fluids or enabling controlled evaporation. Maintaining a moist environment is known to accelerate healing, enhance cell migration and communication, and reduce scarring.^40,41^ A key property contributing to this is the fluid-handling capacity, particularly swelling behavior, which not only balances moisture at the wound site but also facilitates drug release by promoting diffusion as the patch absorbs exudates.^42^

All formulated patches were evaluated for their swelling capacities (Figure 5a). Rapid absorption was observed across all samples, reaching equilibrium within 30 min. Notably, freeze-dried patches demonstrated superior fluid uptake—up to 1100% for GelMgHA@GelChit3:1_freeze and 1300% for GelMgHA@ChitGly2:1_freeze—greatly surpassing the air-dried variant (110%) and the typical recommended range (150–900%).^43^ This enhanced absorption is attributed to their highly porous, sponge-like structure, making them especially suitable for heavily exuding wounds. However, GelMgHA@GelChit3:1_freeze showed a notable decline in swelling after 24 h, suggesting early onset of degradation. Degradation studies in PBS at 37 °C (Figure 5b) revealed that material composition, rather than fabrication method, predominantly governs degradation behavior. Blends containing chitosan and glycerol (GelMgHA@ChitGly2:1_freeze and casting) exhibited the highest stability, with minimal weight loss (especially in the DHT-cross-linked freeze-dried patch, which degraded only ∼13% over 7 days). Despite some mass loss, these patches maintained their structural integrity, likely due to the leaching of glycerol rather than true polymer degradation.^44,45^ In contrast, the gelatin–chitosan-based GelMgHA@GelChit3:1_freeze patch degraded rapidly, losing 30% of its mass within 24 h and an additional 20% by day 3—eventually transitioning into a soft hydrogel state that limits handling. This underscores the stabilizing role of glycerol as a secondary cross-linker in the chitosan matrix, enhancing the durability of GelMgHA@ChitGly2:1_freeze, even when processed under identical DHT conditions.^33,46^

**Figure 5:**
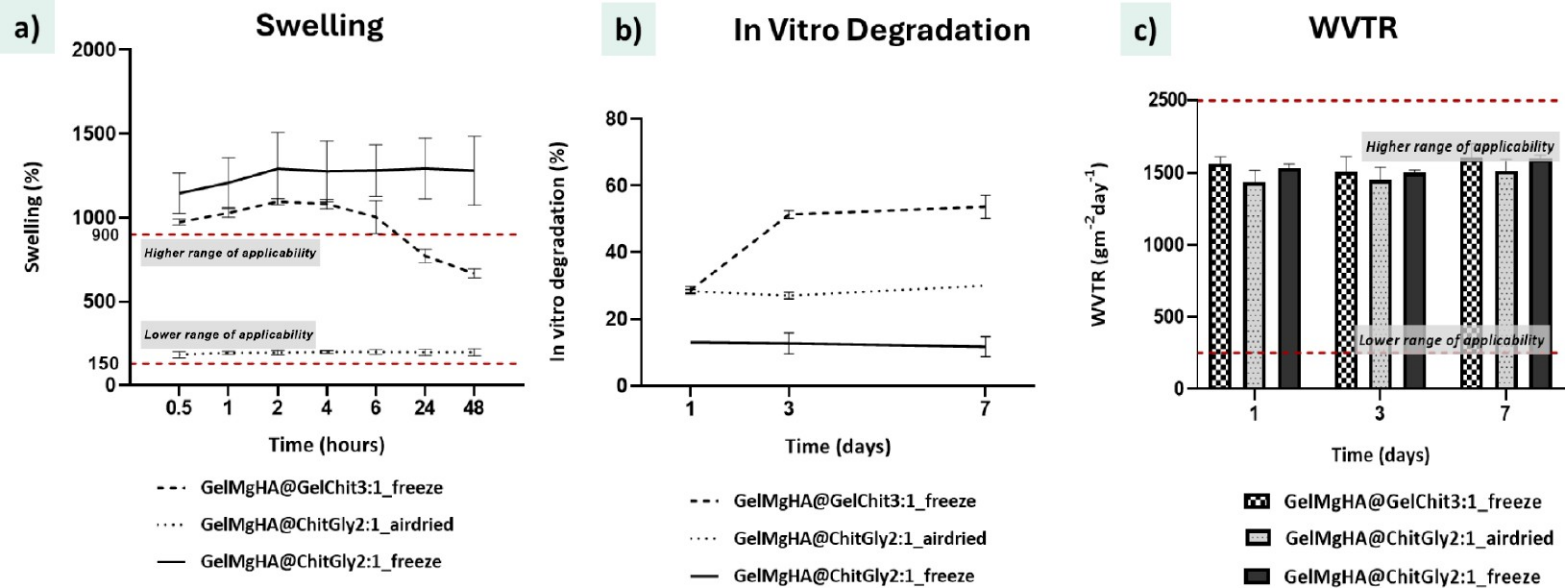
Physical evaluation of hybrid single-layer patches. a) Swelling profiles reporting the water uptake percentages of single patches in PBS solution at 37 °C within 48 h; b) degradation curves. Weight loss percentages of single patches in PBS solution at 37 °C within 7 days; c) water vapor transmission rate of single patches at 1, 3, and 7 days.

The water vapor transmission rate (WVTR), another critical parameter for wound healing, was also evaluated (Figure 5c).^40,47^ All patches exhibited WVTR values around 1500 g/m^2^/day, well within the ideal range (250–2500 g/m^2^/day) for dressings. These values remained stable over 1, 3, and 7 days, indicating consistent performance in humid environments and confirming the materials’ suitability for maintaining a moist wound milieu, independent of composition or fabrication method.

### Vancomycin Loading and Release from Single-Layer Patches

Given the complex physiopathology of chronic lesions and the plethora of different comorbidities associated with them, the possibility to offer a personalized care, carefully catered toward the specific needs of the patient, represents a crucial feature for advancing the treatment of chronic wounds. A promising approach involves developing a rapid and effective method for treating patches with commercially available therapeutic agents.

Taking advantage of the patches’ swelling properties discussed above, attributed to their hybrid porous structure, we studied an antibiotic loading protocol performed by absorption so that it can be easily reproduced in a hospital setting immediately prior to the wound medication. Vancomycin hydrochloride (VNC) was chosen as the test drug due to its broad efficacy against Staphylococcus spp., and its use in treating infected wounds in hospitalized patients, especially under suspicion of methicillin-resistant S. aureusinfection.^48^ To ensure the total absorption of drug solutions and precise drug loading, the medium volume that each sample was able to absorb was measured beforehand. The drug was dissolved in PBS to its solubility limit (50 mg/mL), and the selected volume of the solution was dripped onto the scaffold. The amounts of VNC to be loaded on the scaffold were selected, taking into account both the minimum inhibitory concentrations (MIC) for VNC (≤2 mg/L)^36^ and the working limits of the experimental setup. Therefore, 1.5 mg of the drug was loaded into each scaffold to ensure that drug concentrations in the release medium exceeded the detection limit of the UV–vis spectrophotometer and reached the MIC required for an inhibitory effect. The drug incorporation yield was assessed by using UV–visible spectroscopy. After scaffold loading, a known volume of PBS was added to the wells where the loading had taken place to collect any unabsorbed solution left on the walls. The concentrations of the solutions were then determined, and the actual amount of drug absorbed in each scaffold was estimated by subtracting the calculated ideal loading. The loading results are reported in Table 1. As the data show, the selected adsorption loading method proves highly effective for the patches obtained through freeze-drying. These patches, characterized by their highly porous structure and superior swelling ability, enable almost complete drug loading. In contrast, the notably inefficient drug loading observed for the air-dried patches (less than 20% compared with the calculated quantity) aligns with expectations. This inefficiency is associated with their low-porosity nature, rendering them among the three analyzed samples with the least favorable loading performance.

**Table 1:** Loading Efficacy of the Patches with VNC

Code	% Loading media	% Loading SD
GelMgHA@GelChit3:1_freeze	96.2%	0.7%
GelMgHA@ChitGly2:1_casting	18.7%	7.9%
GelMgHA@ChitGly2:1_freeze	99.1%	0.8%

Release tests were performed in PBS at 37 °C under constant and slow oscillation to better reproduce the physiological conditions. At specific times (from 0 to 7 days), 10% of the total PBS volume was collected and replaced with the same amount of fresh PBS solution every time. This approach was deliberately chosen to maintain dynamism in the elution environment, resembling the natural exchange of physiological fluids during the processes of wound healing and regeneration. The experiment was monitored for 168 h (7 days), and the collected measurements were elaborated to obtain the release curves reported in Figure 6.

**Figure 6:**
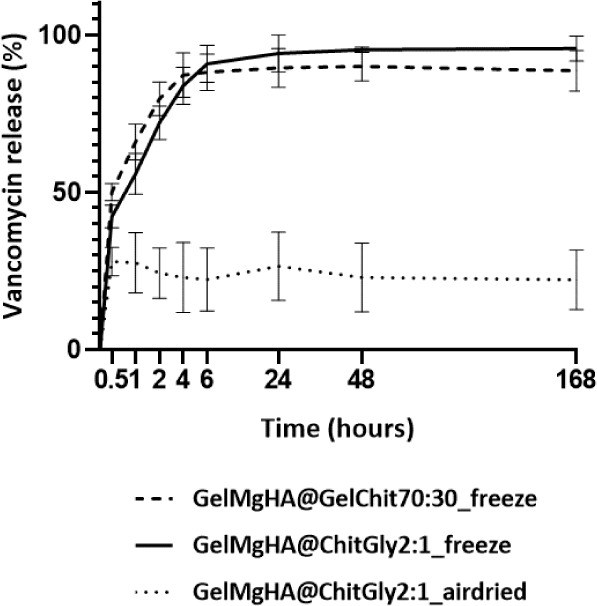
VNC kinetic release from hybrid single-layer patches registered through the UV–visible technique in PBS at 37 °C.

The release kinetics highlight a quite different trend between the porous patches obtained through freeze-drying and the air-dried patch. In the case of the freeze-dried patches, there was a rapid and nearly complete release of VNC within the first 6 h, accounting for approximately 90% of the drug released from both GelMgHA@GelChit3:1_freeze and GelMgHA@ChitGly2:1_freeze, followed by a slower VNC release over subsequent days. Conversely, the air-dried patch, on top of an already insufficient loading (Table 1), exhibited a drug release challenging and inefficient, with only about 20% of the drug released. Furthermore, this release occurred entirely within the first half-hour, with no further release observed over the following days. These findings suggest that the air-dried patch (GelMgHA@ChitGly2:1_casting) may not be suitable for achieving effective and controlled drug release at the infection site. Moreover, the challenges in both loading and releasing the drug could render the patch dressing procedure ineffective and costly in a hospital setting, potentially leading to significant drug wastage due to the low percentage of drug loading and release.

Having established that the patch GelMgHA@ChitGly2:1_casting is not suitable for VNC loading, we focused our attention on the two freeze-dried patches. Although both patches exhibit a “burst” release behavior, with approximately 90% of VNC released gradually in the first 6 h, when looking at the release curves in detail, slight differences in release behavior between the two compositions can be observed. Specifically, while the blend with gelatin, GelMgHA@GelChit3:1_freeze, reaches the release plateau already after 4 h, the patch composed of chitosan and glycerol gradually increases the release up to 48 h, before reaching the plateau. Furthermore, it can be observed that a complete release of the loaded VNC is not observed for either patch, even at longer times (7 days); however, although the GelMgHA@GelChit3:1_freeze patch terminates its release activity sooner, it retains a higher percentage of VNC within the patch, with 10% not being released even after 7 days, compared to 5% remaining within the GelMgHA@ChitGly2:1_freeze patch. Most importantly, it is worth noticing that the local administration of VNC provided by both patches guarantees a VNC concentration in the PBS medium above the minimum inhibitory concentration (MIC) breakpoints for Staphylococcus spp. (2–4 μg/mL)^36,49^ at every time point, demonstrating that the medicated patches possess the necessary characteristics to perform wound site disinfection while preventing bacterial recolonization. All within the time frame required to act against bacterial colonization at the wound site after debridement, which, for a chronic wound, is around 24–48 h after clinical practice.^50–52^

### Development and Characterization of Hybrid Bilayer Patches

Multilayer wound dressings, consisting of two or more layers, are getting more and more consideration, thanks to their ability to better support the process than single-layer dressing. They can be easily adapted to meet the requirements of wound healing by the choice of chemical and physical properties of the constituent biomaterials, enabling to take advantages of the compositions and intrinsic features of each layer.^53–55^ We chose to take advantages of the two distinct morphologies designed through different casting techniques by developing two bilayered patches combining the air-dried patch (GelMgHA@ChitGly2:1_casting), acting as the upper layer, with the porous, freeze-dried patches (GelMgHA@GelChit3:1_freeze, GelMgHA@ChitGly2:1_freeze) as the bottom layer. This design enables us to capitalize on the characteristics of both morphologies: the upper layer serves as a protective barrier against external bacterial contamination while providing necessary vapor permeability to prevent tissue maceration. Meanwhile, the bottom layer, with its highly porous structure, effectively controls exudate absorption, ensures optimal adhesion to the wound, facilitates cell permeation and efficient loading with a specific drug before dressing application, and expedites its release at the wound site, ensuring both wound disinfection and supporting skin regeneration.

The bilayered patches were obtained by combining the air-dried layer GelMgHA@ChitGly2:1_casting with the freeze-dried layer GelMgHA@GelChit3:1_freeze to obtain the Bilayer-1 (BL-1) or with GelMgHA@ChitGly2:1_freeze to obtain the Bilayer-2 (BL-2), as reported in the Table 6 of Materials and Methods section.

#### Morphological Characterization

Electron scanning microscopy performed on the cross-section of the bilayer patches BL-1 and BL-2 (Figure 7) reveals that two distinct layers could be identified, with a clear distinction between the nonporous structure of the air-dried top layer and the highly porous morphology of the bottom layer. The average pore size of the bottom layer is 121.2 ± 35.6 μm for BL-1 and 113.1 ± 47.9 μm for BL-2, confirming the same trend of pore dimensions observed for the single-layers. Macroporosity, assessed through the water-squeezing method, results in values of 62.4 ± 2.7 for BL-1 and 69.1 ± 2.1 for BL-2. This finding is consistent with our previous observations for single-layers, as BL-2, composed of GelMgHA@ChitGly2:1_freeze as the bottom layer, exhibits higher macroporosity compared to BL-1 (bottom layer GelMgHA@GelChit3:1_freeze). However, the overall macroporosity values show a decrement compared to those of the single-layers GelMgHA@GelChit3:1_freeze and GelMgHA@ChitGly2:1_freeze, attributable to the contribution of the top layer (GelMgHA@ChitGly2:1_casting).

**Figure 7:**
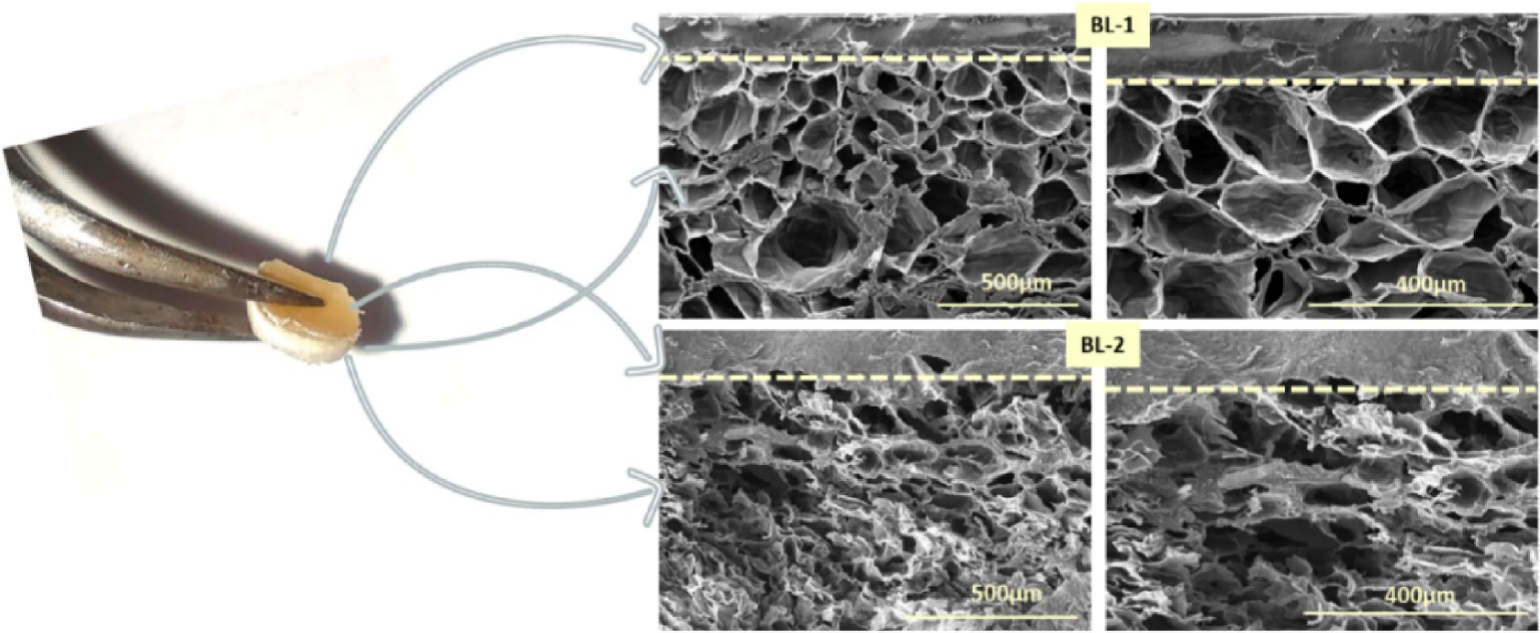
Bilayer patches morphological evaluation. Cross sections of BL-1 and BL-2 observed with SEM were reported in the panel at different magnifications to better appreciate the entire morphology of both patches.

#### Physical Characterization

The bilayered membranes (BL-1 and BL-2) demonstrated promising swelling behavior, degradation profiles, and water vapor transmission rates (WVTR), highlighting their potential for effective wound healing applications. As shown in Figure 8a,b, both bilayer constructs exhibited swelling capacities around 400%, positioning them within the optimal range (150–900%) for chronic wound dressings. Notably, BL-1 absorbed slightly less water than BL-2 but maintained stable swelling over 48 h, unlike its single-layer counterpart, which showed a sharp decline after 24 h, suggesting that the protective upper layer plays a stabilizing role.

**Figure 8:**
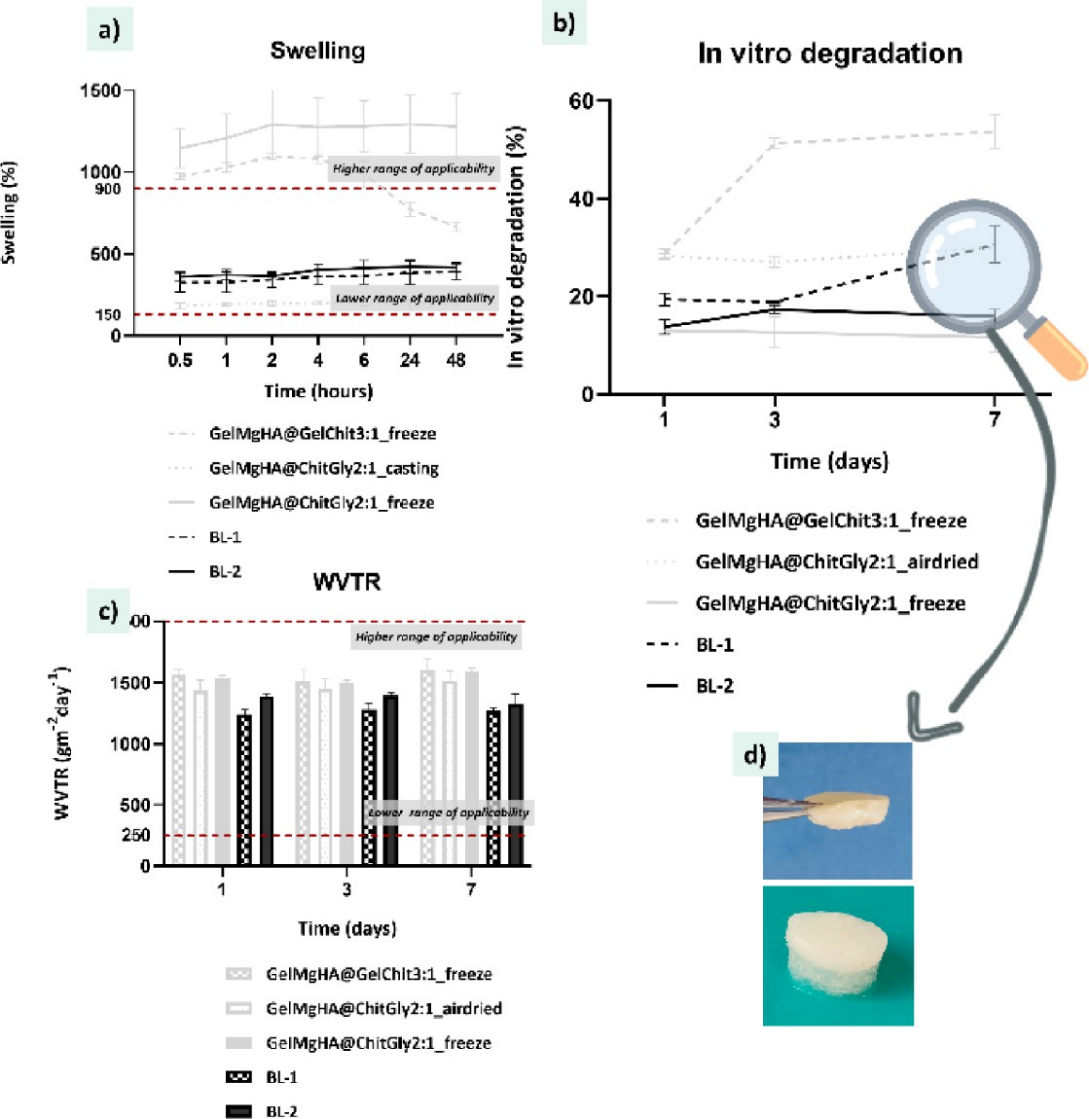
Physical evaluation of BL-1 and BL-2 patch plotted against the constituent single-layer (gray). a) Swelling profiles reporting the water uptake percentages of patches in PBS solution at 37 °C within 48 h; b) degradation curves. Weight loss percentages of single patches in PBS solution at 37 °C within 7 days; c) water vapor transmission rate of single patches at 1, 3, and 7 days; d) macroscopical images of BL-1 (top) and BL-2 (bottom) after 7 days in PBS at 37 °C. No detachment between top and bottom layer was observed for either BL patch; however, partial loss of the 3D structure is visible in the porous bottom layer of BL-1, while the bottom layer of BL-2 remains fully intact.

Regarding degradation (Figure 8b), BL-1 and BL-2 showed intermediate behavior compared with their constituent layers. BL-1 degraded more slowly, avoiding the rapid breakdown observed in its porous bottom layer. This slower degradation supports the hypothesis that the bilayer configuration effectively limits exposure to external stressors. However, by day 7, partial structural collapse of the bottom layer indicated its inherent tendency to revert to a hydrogel state. BL-2, on the other hand, maintained a more stable degradation profile throughout the 7-day period. It is worth noting that the different behavior in terms of degradation exhibited by BL-1 and BL-2 does not determine the superiority of one compared to the other for wound dressing applications, as hydrogels have shown to be of great interest in wound healing. Furthermore, the combination of a hydrogel bottom layer, which can neatly fill up the wound area and degrade inside it, releasing the therapeutics hosted inside the matrix, with a compact upper layer for protection purposes, has already shown promising results.^9^

Interestingly, WVTR values (Figure 8c) for both bilayers were slightly lower than those of the single layers, approximately 1250 g/m^2^/day for BL-1 and 1350 g/m^2^/day for BL-2, likely due to the increased thickness of the bilayered structure. Despite this, both values remain well within the ideal range for wound healing, and their stability over time confirms the membranes’ consistent breathability in humid conditions. Together, these results emphasize how the bilayer design balances absorption, degradation, and vapor permeability, offering a multifunctional platform suitable for a range of chronic wound scenarios.

### Vancomycin Loading and Release from BL Patches

The BL patches were loaded with VNC following the procedure illustrated in Section 3.4. Given our findings indicating superior drug absorption in the spongy bottom layer, loading was focused on this layer. Table 2 presents the loading efficacy for both bilayer configurations, reaffirming the high loading efficiency observed previously for the GelMgHA@GelChit3:1_freeze and GelMgHA@ChitGly2:1_freeze single-layer patches.

**Table 2:** Loading Efficacy of the BL-Patches with VNC

Code	% Loading media	% Loading SD
BL-1	98.47%	0.02%
BL-2	99.88%	0.01%

The release patterns registered for the bilayered patches BL-1 and BL-2 were plotted against the curves obtained for the corresponding single-layer patches GelMgHA@GelChit3:1_freeze and GelMgHA@ChitGly2:1_freeze, which constitute the porous bottom layer of each bilayered patch (respectively, BL-1 and BL-2), as depicted in Figure 9.

**Figure 9:**
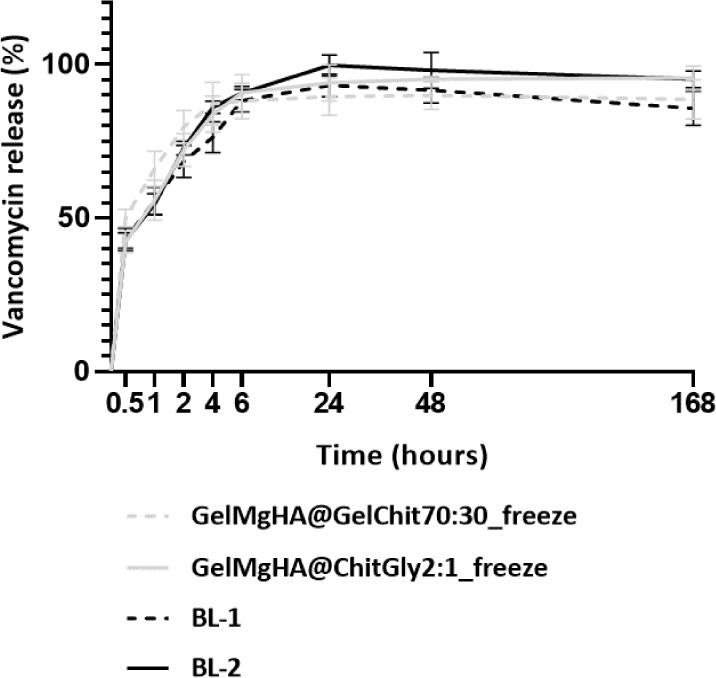
VNC release from bilayer patches plotted against the release curve obtained for single-layers GelMgHA@GelChit3:1_freeze and GelMgHA@ChitGly2:1_freeze (gray).

As illustrated from the curves, the release kinetics of VNC for BL-1 and BL-2 closely mirror those observed for GelMgHA@GelChit3:1_freeze and GelMgHA@ChitGly2:1_freeze, with 91% and 96% of VNC released by day 7, respectively. This indicates that the bilayer structure does not significantly alter the drug absorption and release capabilities of the porous bottom layer. Moreover, the trend observed in the single-layer patches, wherein more VNC remains embedded in the BL-1 matrix compared to BL-2 at day 7, is also confirmed.

### Antimicrobial Evaluation

#### In Vitro Assessment of Patch Antimicrobial Activity

The antibacterial activity of the fabricated patches loaded with VNC was evaluated by using two different methodologies to assess (i) their effectiveness in inhibiting bacterial proliferation immediately after the application at the site of infection and (ii) their prolonged antibacterial effect up to 3 days from the use. Table 3 and Figure S1 report the disk diffusion test results.

**Table 3:** Antibacterial Activity^a^

Samples	S. aureus ATCC 25923	S. epidermidis ATCC 12228
GelMgHA@GelChit3:1_freeze	19–22	22–27
GelMgHA@ChitGly2:1_freeze	23–24	24–26
GelMgHA@ChitGly2:1_casting	19–22	18–25
BL-1	20–22	21–25
BL-2	21–23	22–24
VCN^b^	21–23	25–28

a Diameter of the inhibition zone (range in millimeters) against ATCC reference strains.

b Sterile paper disk loaded with VNC.

All samples displayed inhibitory activity against both reference strains, and no differences were observed between antibiotic-loaded patches and the sample control containing the same amount of VNC. This finding suggests that the VNC can diffuse through the agar, maintaining its potency toward the tested bacteria, and that all patches are suitable to preserve the antibiotic drug activity.

In addition, the antibacterial activity of VNC released from the patches in a liquid solution was evaluated in vitro, following different incubation intervals to determine the inhibitory potential over time. Figure 10 shows representative results of the microdilution broth assays for samples analyzed at 30 min, 24 h, and 72 h toward S. aureus. As depicted, all samples after 30 min of incubation in PBS displayed a strong and immediate inhibitory activity on bacterial growth (see 1:10 dilution); samples recovered at 24 h inhibited bacterial proliferation, with the only exception for GelMgHA@ChitGly2:1_casting that completely lost its potency; samples collected following 3 days of incubation in the liquid solution-maintained activity but at different extent. In particular, among the single-layer patches, GelMgHA@GelChit3:1_freeze reduced S. aureus growth by 57.7% compared to the positive control, and remarkably, the BL-1 still retained its potency; the samples being the samples with the most prolonged activity.

**Figure 10:**
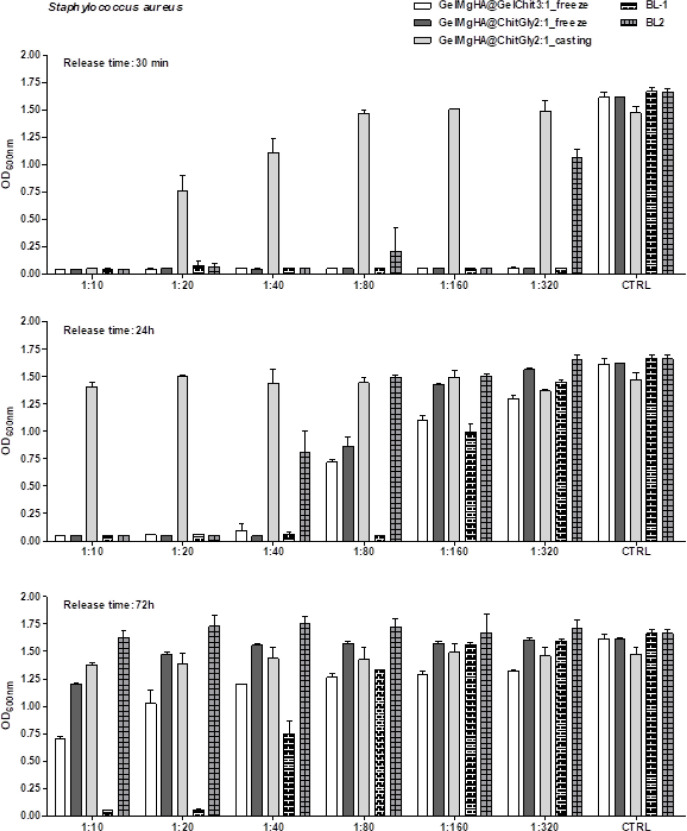
Antibacterial activity against S. aureus of the VNC released in PBS solutions at different time intervals from the patches. Data are the means ± SD of the optical density at 600 nm. CTRL, positive growth controls.

The microdilution broth assay was also performed against S. epidermidis (Figure S2) and, as expected, the results were comparable to those observed with S. aureus, confirming the susceptibility to VNC for the most relevant Staphylococci for human infections.

These results were confirmed by measuring the residual antibacterial activity of the VNC-loaded patches after 3 days of incubation. As detailed in Table 4 and Figure S3, results of the disk diffusion assay demonstrated that GelMgHA@ChitGly2:1_casting was the single-layer sample with the lower inhibitory activity against S. aureus and failed to inhibit S. epidermidis proliferation. The bilayer GelMgHA@GelChit3:1_freeze, despite the prolonged release of VNC in the solutions, preserved a strong inhibitory activity, thus ensuring high effectiveness against Staphylococci.

**Table 4:** Antibacterial Activity of the Patches after Release in PBS Solution^a^

Samples	S. aureusATCC 25923	S. epidermidisATCC 12228
GelMgHA@GelChit3:1_freeze	18–19	20–21
GelMgHA@ChitGly2:1_freeze	13–15	11–12
GelMgHA@ChitGly2:1_casting	8–9	NA^∧^
BL-1	18–19	20–21
BL-2	11–12	11–12

a Diameter of the inhibition zone (range in millimeters) against ATCC reference strains. [^∧^NA: not appeared]

Overall, these findings clearly demonstrated the greater antibacterial activity of BL-1 hybrid patch compared to all others; indeed, it released a quantity of VNC such as to be inhibitory for a prolonged period of time, and the patch itself preserved a potent effectiveness against both Staphylococcal strains.

#### Scanning Electron Microscopy

A direct visualization of the cellular damages induced by the bilayer patches was evaluated by SEM. Figures 11 and S4 show the images of the reference ATCC strains incubated for 6 h at 37 °C on the VNC-loaded and unloaded samples, as controls. Bacterial cells on the untreated samples appeared with bright and smooth surfaces, completely covering the materials. Conversely, on VNC-loaded patches, cells presented large lesions and ruptures in the cell membranes, and the cell surface was significantly modified, being rough and corrugated. In addition, a lower overall number of cells was observed on the treated patches compared to the controls.

**Figure 11:**
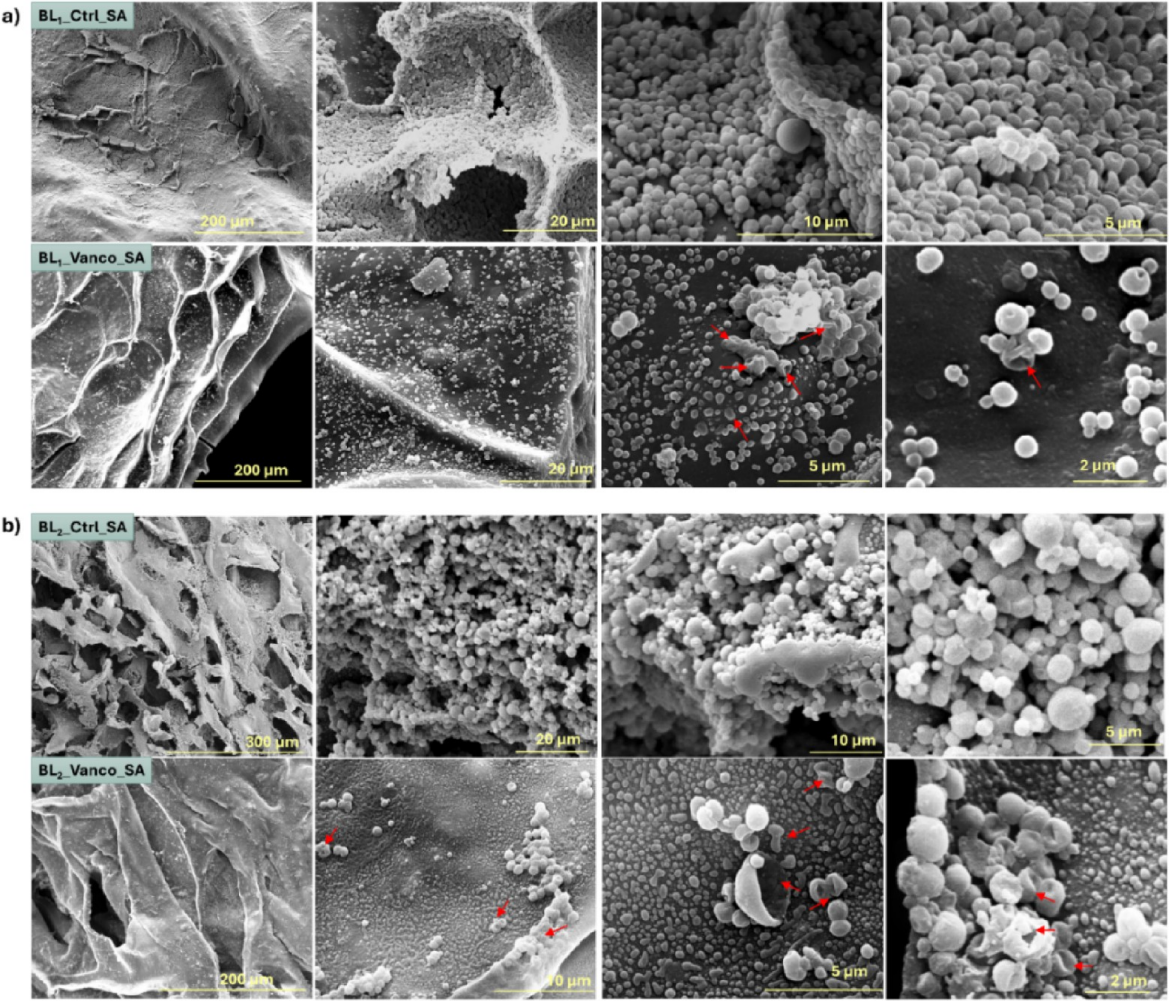
Scanning electron microscopy (SEM) analysis of BL-1 and BL-2 samples both unloaded and loaded with VNC, after 6 h of incubation with S. aureus (SA). a) The images refer to BL-1, with unloaded samples at the top and samples loaded with VNC at the bottom. b) The images refer to BL-2, with unloaded samples at the top and samples loaded with VNC at the bottom. Red arrows indicate bacteria with evident alterations of the membrane in the VNC-treated patches.

### In Vitro Cytocompatibility Assay

The cell viability and morphological analysis of WS1 cells seeded into the different patch types were assessed over a period of 7 days to evaluate cell behavior and confirm the absence of toxicity. Tests were conducted both on the single-layer patches independently to demonstrate their ability to support cell viability and proliferation, and on the freeze-dried patches GelMgHA@GelChit3:1_freeze and GelMgHA@ChitGly2:1_freeze, after loading them with Vancomycin and subsequently unloading in cell culture medium for 24 h. This was done to evaluate whether the patches retained their cytocompatibility following drug release.

The qualitative Live/Dead assay demonstrates a high ratio of live cells in all samples after 3 days of culture. Cell density is consistent in all groups except the GelMgHA@ChitGly2:1_freeze sample, which appeared to have slightly lower cell colonization. Morphological evaluation confirms the presence of well-spread cells in all samples, with no significant signs of cell damage (Figure 12). However, due to high autofluorescence, it was not possible to analyze the GelMgHA@ChitGly2:1_casting patch by fluorescent microscopy.

**Figure 12:**
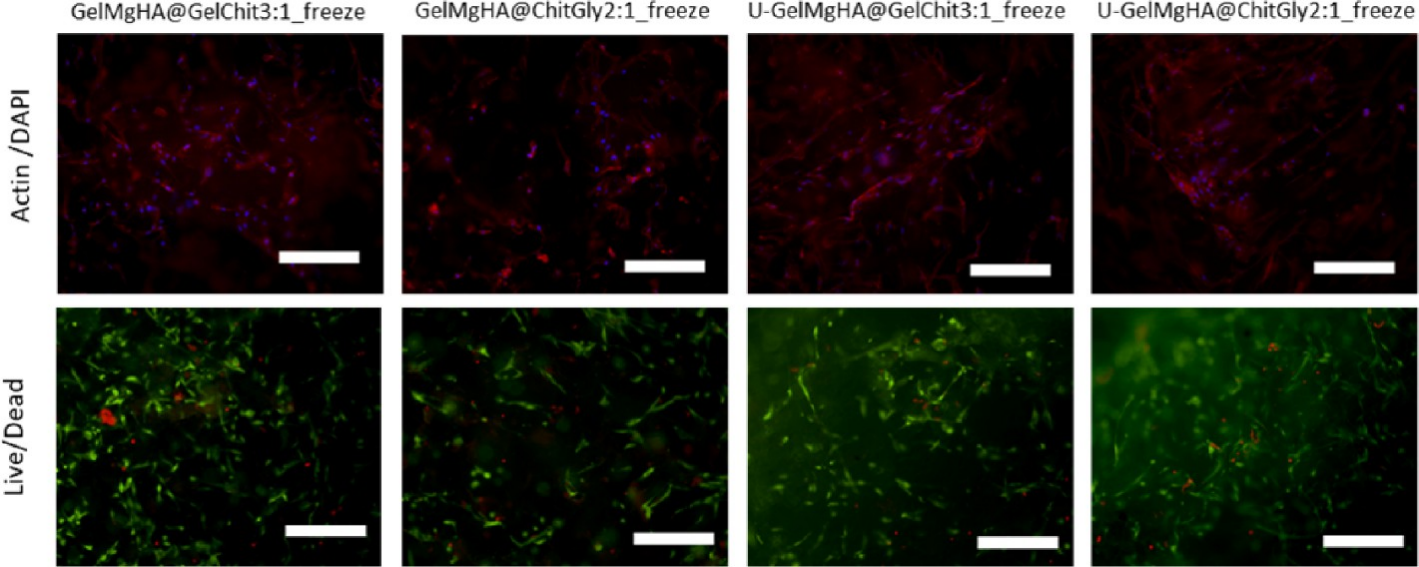
Live and dead and morphological analyze. Representative qualitative images of WS1 cells stained with Actin/DAPI and Live/Dead assays on day 3 postseeding. Images for four patches are shown, excluding GelMgHA@ChitGly2:1_casting, which exhibited high background staining, making interpretation difficult. Scale bar: 300 μm.

Quantitative cell proliferation analysis (Figure 13) indicates a significant increase in cell viability over time in the patch GelMgHA@GelChit3:1_freeze (d1 vs d7 and d3 vs d7, *p* < 0.0001), GelMgHA@ChitGly2:1_casting (d1 vs d7 and d3 vs d7, *p* < 0.0001), and U-GelMgHA@GelChit3:1_freeze (d1 vs d3, *p* < 0.01 and d3 vs d7, *p* < 0.0001). In contrast, the sample GelMgHA@ChitGly2:1_freeze shows no significant cell proliferation, while the patch U-GelMgHA@ChitGly2:1_freeze exhibits an initial increase on day 3 (*p* < 0.01), followed by a marked decrease by day 7 (*p* < 0.01).

**Figure 13:**
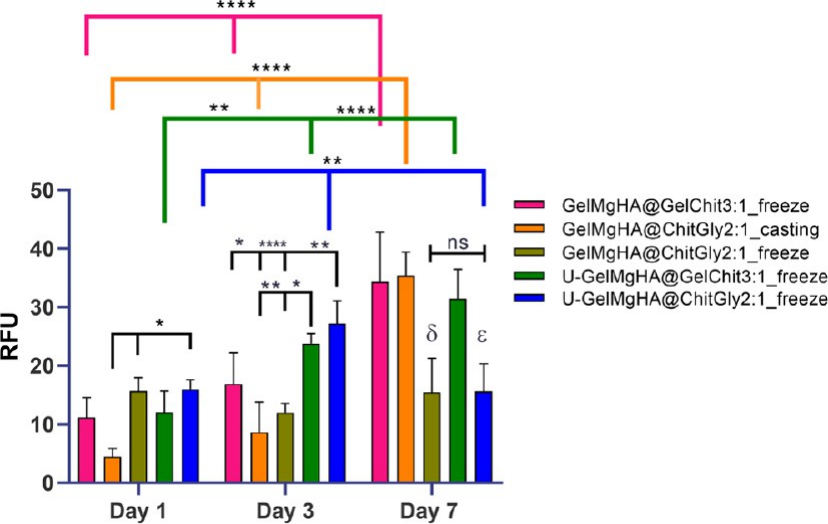
Cell proliferation by PrestoBlue assay at days 1, 3, and 7. The RFU data (mean ± SEM) are presented in the graph. Statistical analysis using two-way ANOVA with Tukey’s multiple comparisons test indicate in the graph: *p < 0.05, **p < 0.01, and ****p < 0.0001. Symbols δ and ε represent significant differences for GelMgHA@ChitGly2:1_freeze and U-GelMgHA@ChitGly2:1_freeze, respectively, compared to the other samples, with δ: **** with GelMgHA@GelChit3:1_freeze and GelMgHA@ChitGly2:1_casting, and *** with U-GelMgHA@GelChit3:1_freeze, and ε: **** with GelMgHA@GelChit3:1_freeze and GelMgHA@ChitGly2:1_casting, and *** with U-GelMgHA@GelChit3:1_freeze.

Looking in detail at the effect on cell viability induced by the different sample types, it is possible to observe that GelMgHA@ChitGly2:1_casting induces statistically significant lower cell viability on Day 1 compared to GelMgHA@ChitGly2:1_freeze and U-GelMgHA@ChitGly2:1_freeze (*p* < 0.05). This could potentially be attributed to the lower cell seeding efficiency of GelMgHA@ChitGly2:1_casting, which lacks the porous structure necessary for the initial cell adhesion.^56^

By Day 3, patches U-GelMgHA@GelChit3:1_freeze and U-GelMgHA@ChitGly2:1_freeze showed statistically significant high cell viability. In detail, the viable cells grown on U-GelMgHA@GelChit3:1_freeze are statistically significantly higher compared to GelMgHA@ChitGly2:1_casting and GelMgHA@ChitGly2:1_freeze (*p* < 0.05 and *p* < 0.01, respectively), while U-GelMgHA@ChitGly2:1_freeze shows higher cell viability compared to GelMgHA@GelChit3:1_freeze, GelMgHA@ChitGly2:1_casting, and GelMgHA@ChitGly2:1_freeze (*p* < 0.05, *p* < 0.0001, and *p* < 0.01, respectively).

On Day 7, GelMgHA@ChitGly2:1_freeze and U-GelMgHA@ChitGly2:1_freeze showed statistically significantly lower cell viability than the other groups (Figure 13). These results suggest a patch-dependent effect on WS1 cell viability, with GelMgHA@GelChit3:1_freeze, GelMgHA@ChitGly2:1_casting, and U-GelMgHA@GelChit3:1_freeze supporting greater long-term viability compared to GelMgHA@ChitGly2:1_freeze and U-GelMgHA@ChitGly2:1_freeze.

The higher cell viability observed in samples GelMgHA@GelChit3:1_freeze and U-GelMgHA@GelChit3:1_freeze could be partly explained by the higher presence of gelatin in the porous structure, which is well known to improve the biocompatibility of the scaffold and, when used on combination with other polymers or bioactive factors, can promote cellular adhesion, differentiation, proliferation, and cell-scaffold interactions.^57^

However, the results demonstrated that the presence of chitosan and glycerol can also support cell proliferation, but in the case of this composition, the casting protocol appears to play a role in the cellular response observed for GelMgHA@ChitGly2:1_casting and GelMgHA@ChitGly2:1_freeze. In fact, GelMgHA@ChitGly2:1_casting, despite an initial decrease in cell adhesion, showed better long-term cell viability compared to GelMgHA@ChitGly2:1_freeze.^10,58,59^

Finally, it is possible to assert that the loading of the drugs does not affect the bioactivity of the patches. In fact, after unloading, both U-GelMgHA@GelChit3:1_freeze and U-GelMgHA@ChitGly2:1_freeze induce similar cellular behaviors compared to their unloaded counterparts, GelMgHA@GelChit3:1_freeze and GelMgHA@ChitGly2:1_freeze.

As a last remark, the suitability of all the patches to support cell viability is further confirmed by SEM analysis of the patches following cell seeding (Figure S5), showing cells well spread and integrated within the surface material with no evident alteration.

### In Vivo Studies on Medicinal Leeches

It is known that the use of animal models in biomedical research is crucial, as it provides a fundamental approach to study and extrapolating to humans the complex cellular and molecular interactions that occur in living tissues. However, the number of animal species that can be used for biomedical experimentation is currently under extensive review due to ethical considerations, stricter controls, and legislative interventions aimed at improving animal welfare. Indeed, the recent Directive 2010/63/EU of the European Parliament and of the Council and the more restrictive Italian DL 26/2014 focused on the “protection of animals used for scientific purposes” strongly promote the use of alternative animal models to vertebrates in biological research. In response to these restrictions, we propose the medicinal leech Hirudo verbana as an excellent in vivo alternative to vertebrate models, such as mice, rats, or rabbits, for developing a novel assay based on a combined system (animal model/biopolymer) to study wound healing and tissue repair. Although leeches are invertebrates, many fundamental cellular and molecular mechanisms of tissue regeneration are conserved across species. Investigating scaffold interactions within this robust regenerative model may yield valuable insights applicable to the development of regenerative therapies in more complex organisms, including mammals.

Our study primarily investigates the early cellular responses to scaffold implantation and the regenerative dynamics in this highly regenerative model. While our focus is on acute wound healing, we acknowledge that H. verbana does not fully recapitulate the pathological features of chronic wounds, such as sustained inflammation, impaired angiogenesis, and bacterial biofilm formation. However, the use of the leech model provides a valuable alternative to reduce the number of tests required in chronic wound models, such as diabetic (*db*/*db*) mice or ischemic wounds, and can offer preliminary insights that help in better assessing the potential for chronic wound applications.

Indeed, in medicinal leeches, the processes underlying wound healing are rapid and characterized by the same phases (angiogenesis, fibroplasia, and remodeling) described for vertebrates.^60,61^ As previously described,^62,63^ following tissue damage, wound healing is achieved by the formation of a pseudoblastema formed by myofibroblasts-like cells.^62,64^ These cells derive from vasocentral cells, typical of leeches and share several similarities with the vertebrate fibrocytes.^65,66^ These vasocentral cells contribute to wound healing by differentiating into myofibroblasts and promoting the contractile force of wound closure. Like vertebrate fibrocytes, vasocentral cells differentiate into myofibroblasts, are characterized by prominent cell surface projections, and express the CD154 marker,^62^ also called CD40 ligand or CD40L. This transmembrane protein, as in vertebrates,^67^ plays a crucial role in wound healing, being implicated in myofibroblast activation. Based on our previous results, we were interested in characterizing the cells infiltrating the scaffolds used to promote faster wound healing. To this aim, we performed morphological and immunofluorescence analyses on injured leech tissue grafted with the following layers: the two freeze-dried single-layers GelMgHA@ChitGly2:1_freeze and GelMgHA@GelChit3:1_freeze, constituting the bottom layer of the bilayered patch, and both the bilayers BL-1 and BL-2.

The following observations can be made from the experiments: starting with the single-layer GelMgHA@ChitGly2:1_freeze (ML), the scaffold was well integrated into the leech tissue. A clot of vasocentral cells surrounding the implanted patch was already visible 72 h after transplantation (Figure 14a). Within 7 days, the wound had closed, and the grafted patch was fully surrounded by vasocentral cells (Figure 14d). These cells, originating from the leech tissue surrounding the scaffold, colonized the single-layer and adopted a spindle shape, with thin cytoplasmic projections adhering to the scaffold trabeculae (Figure 14b,e). Immunofluorescence assays (Figure 14c,f) confirmed the presence of CD154+ cells colonizing the single-layer. The number of CD154+ cells increased over time, following the graft. Specifically, 72 h after implantation (Figure 14c), CD154 expression was observed in the peripheral area of the GelMgHA@ChitGly2:1_freeze patch in direct contact with the leech tissue. After 7 days, numerous spindle-shaped CD154+ cells were detected in the deeper regions of the patch (Figure 14f).

**Figure 14:**
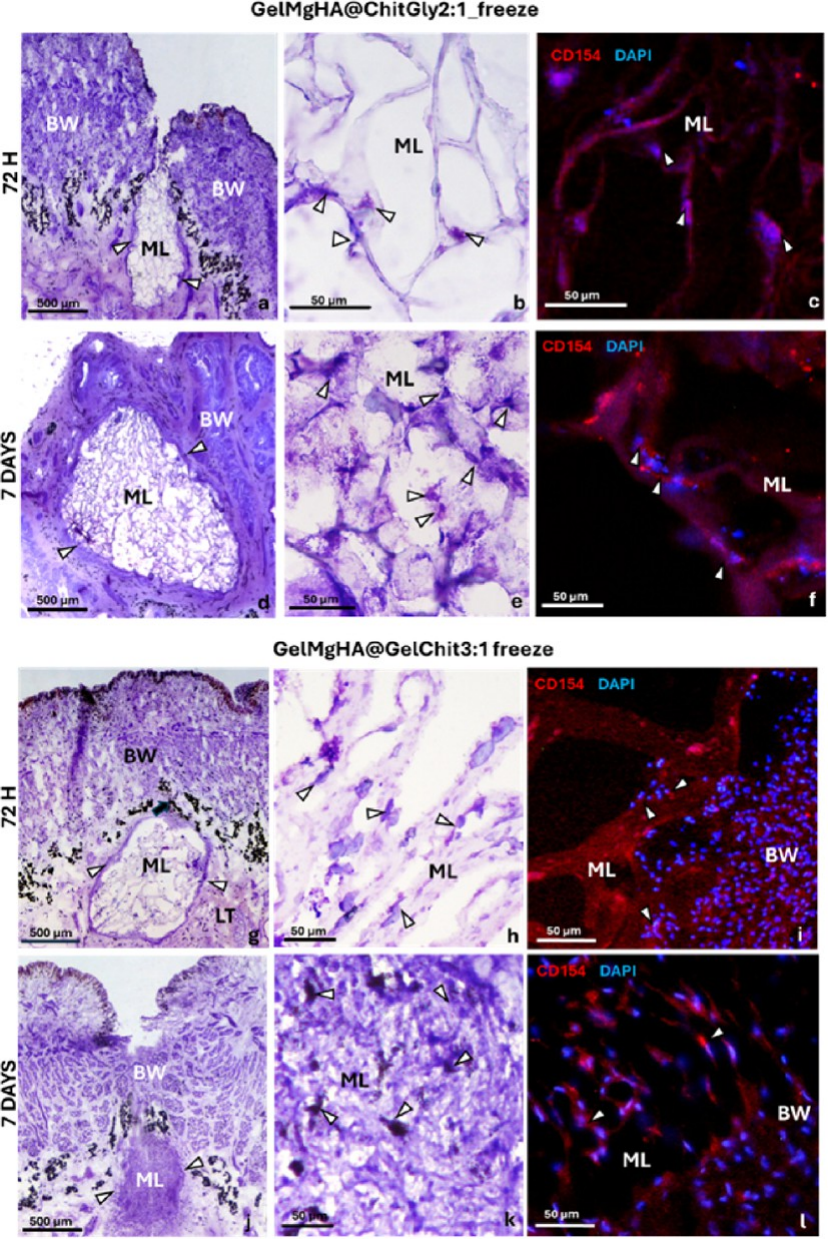
Cross-section of a leech body wall (BW) grafted with a monolayer of GelMgHA@ChitGly2:1_freeze (ML) (a–f) and GelMgHA@GelChit3:1_freeze (ML) (g–l) after 72 h and 7 days postimplantation. A compact layer of vasocentral cells stained with VF (arrowheads) surrounds the ML (arrowheads in a,d,g,j). Details of vasocentral cells (arrowheads) colonizing the ML (b,e,h,k). Immunofluorescence assay reveals CD154+ cells (red in c,f,i,l) colonizing the ML, with cell nuclei stained in blue using DAPI.

Moving on to the second single-layer patch, GelMgHA@GelChit3:1_freeze, this patch also integrated well into the leech tissue (Figure 14g–l). After 72 h of implantation, CD154+ vasocentral cells formed a thin, yet continuous layer surrounding the peripheral region of the monolayer, progressively colonizing it (Figure 14g,h). Notably, by 7 days postimplantation (Figure 14j–l), the patch was densely populated with CD154+ vasocentral cells. This finding was consistent with in vitro results, where GelMgHA@GelChit3:1_freeze demonstrated excellent cell viability and promoted the proliferation of WS1 fibroblast cells isolated from human skin (Figure 13). However, it is important to note that its 3D structure was altered. As highlighted in Physical Evaluation section, GelMgHA@GelChit3:1_freeze exhibited faster degradation kinetics compared to the other single-layer patch, GelMgHA@ChitGly2:1_freeze. Specifically, the patch lost its 3D shape after immersion in water for more than 24 h, transitioning into an almost hydrogel-like state.

This hypothesis was further confirmed once the experiments on the BL-1 (Figure 15a–f) and BL-2 (Figure 15g–l) bilayers were carried out. In fact, in accordance with what was obtained for the single-layer GelMgHA@ChitGly2:1_freeze, the results for BL-2 (top layer: GelMgHA@ChitGly2:1_casting; bottom layer: GelMgHA@ChitGly2:1_freeze) show that, after 72 h from bilayer grafting, the bottom layer appears well integrated with the leech tissues through a thin layer of vasocentral cells surrounding the peripheral region of the scaffold (Figure 15a). Some elongated (Figure 15b) and CD154+ (Figure 15c) vasocentral cells were visible, adherent to the bilayer trabeculae. In contrast, the top layer did not appear to integrate well into the leech tissue, as demonstrated by large gaps between the vasocentral cell coat and the top layer (Figure 15a). Furthermore, the top layer was not colonized by spindle-shaped CD154+ vasocentral cells (Figure 15a–c).

**Figure 15:**
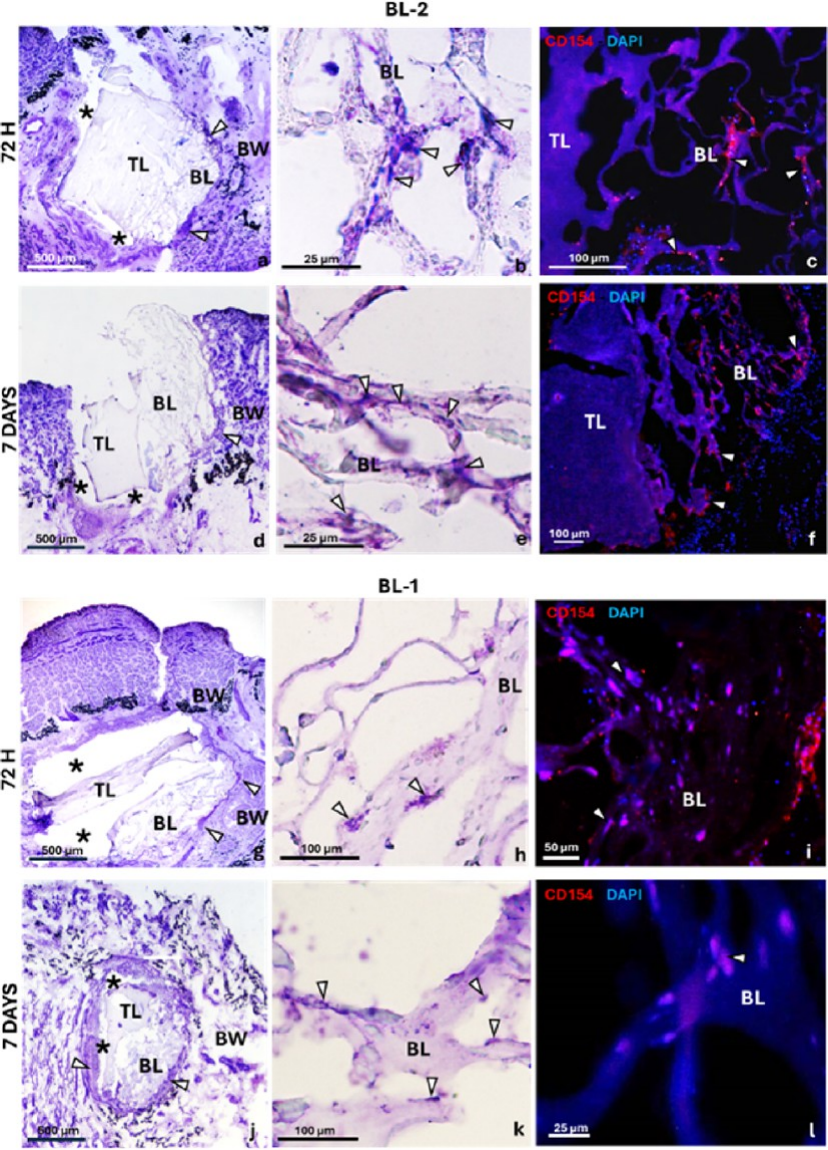
Cross section of a leech body wall (BW) grafted with bilayer BL-2 (a–f) or BL-1 (g–l) after 72 h and 7 days from implantation, stained with violet and fuchsin (VF). The bottom layer (BL) is coated by a thin layer of vasocentral cells (arrows in a,d,g,j). The top layer is separated from the leech tissue by a large gap (* in a,d,g,j). Panels (b,e,h,k) show details of the BL, colonized by spindle-shaped vasocentral cells stained with VF. Panels (c,f,i,l) display immunofluorescence staining of vasocentral cells expressing CD154 (in red) as they infiltrate the BL. Nuclei of cells are stained in blue with DAPI (d,h).

After 7 days, the wound was still open. The top layer was completely detached from the leech tissues, while the bottom layer appeared peripherally coated by a thicker layer of vasocentral cells (Figure 15d). A greater number of vasocentral cells (Figure 15e) and CD154+ (Figure 15f) were colonizing the bottom layer trabeculae.

As previously described for BL-2, in the case of BL-1, the top layer did not integrate with the leech tissues and was not colonized by cells (Figure 15g,j). In contrast, the bottom layer was surrounded by a thick layer of vasocentral cells (Figure 15h,k) and was infiltrated by CD154+ vasocentral cells (Figure 15i,l), confirming the cytocompatibility of the single-layer GelMgHA@GelChit3:1_freeze, as observed earlier. No signals were detected in the negative control immunofluorescence assays, in which the primary antibodies were omitted (Figure S6).

Finally, a remark on the fact that the top layer of the bilayer patch is not integrated into the leech tissue. One of the problems associated with chronic wound care is the need for frequent dressing changes and the tendency of traditional dressings to adhere to the wound, causing secondary damage and pain to the patient when removed. In this context, the design of a bilayer dressing, in which the lower layer can be fully colonized by host tissue cells, providing regenerative stimulation and local delivery of an antibiotic, while the upper layer, which provides protection against microbial invasion, spontaneously detaches as the integration of the lower layer progresses, may offer a viable alternative. In fact, this design allows one to combine both the need to ensure a favorable environment for wound healing and the need to avoid damaging the newly formed tissue when changing the dressing.

## Conclusions

This study led to the development of novel formulations of biomimetic and bioactive hybrid patches for the management of chronic wounds. They are developed with a bilayer design to simultaneously exert wound protection, infection treatment, and dermal tissue regeneration. The bilayer structure, comprising an air-dried membrane as the top layer and a freeze-dried porous bottom layer, demonstrated the potential of exploiting distinct morphologies to obtain biomaterials with multiple functionalities. Inspired from the distinct layers composing the skin tissue (epidermal and dermal), were optimized a compact upper layer acting as a protective barrier against external contaminants while allowing vapor permeability, essential for maintaining an optimal wound environment, and a highly porous bottom layer that facilitates exudate absorption, promotes adhesion and permeation of local cells, and enables further functionalization with drugs by absorption. The regenerative stimuli were imparted with the integration of bioresorbable GelMgHA hybrid particles, which are suitable for releasing bioactive Mg^2+^ ions at the wound site, crucial to influence the migration and adhesion of human skin fibroblasts and promote angiogenesis. The developed drug-loading protocol was specifically designed to be carried out in a medical setting to exert personalized care and, at the same time, provide an effective inhibitory potential for up to 3 days directly at the site of bacterial infection.

In conclusion, both formulations demonstrated excellent abilities in driving cell proliferation and tissue regeneration, assessed both in vitro with WS1 fibroblast cells isolated from human skin, and in vivo on a medicinal leech model (H. verbana), as well as being able to provide excellent management of microbial infection, behaving as platforms capable of being tailor-made, medicated, and ensuring sustained local drug release toward personalized therapeutic approaches. Despite the exceptional performance of the patches developed here, we are aware that our study, being at an early stage, has some limitations. In fact, the cellular response studied both in vitro and in vivo with the medicinal leech model was performed with standard models, which do not consider the typical clinical features of chronic wounds, such as impaired angiogenesis, prolonged inflammation, and biofilm formation. Similarly, the role of magnesium ion in skin tissue regeneration has not been specifically studied. These aspects will be addressed in follow-up work as we continue to study and advance our patches.

## Materials and Methods

### Materials

Type A pig skin gelatin (Gel) in powder form (mesh 4, bloom 280) was purchased from Italgelatin (Cuneo, Italy) and used to prepare an aqueous solution (12 wt %) at 40 °C. Low molecular weight chitosan (CS, MW 50000–190000 Da, based on viscosity; deacetylation degree >75%; viscosity 20–300 cps) was supplied by Merck (MO, United States) and used to prepare an aqueous acid solution (2–3 wt %) dissolving Chit into an acetic acid solution (1 wt %). Glycerol (Gly, MW 92.09 g/mol, density 1.25 g/mL) and phosphate buffer saline (PBS) were supplied by Merck (MO, United States), and common high-purity chemical reagents were purchased from Sigma-Aldrich. Vancomycin hydrochloride was obtained from Sigma-Aldrich-Merck (Darmstadt, Germany). Ultrapure water (0.22 mS, 25 °C) was used for the synthesis.

Fetal Bovine Serum (FBS), Minimum Essential Medium α (MEM α A1049001), 1% Penicillin/Streptomycin solution (pen/strep, 100 U/mL-100 μg/mL), and Trypsin-EDTA 0.5% no phenol red (10× trypsin) were supplied by Gibco (Life Technologies, Thermo Fisher Scientific, Waltham, Massachusetts, USA). PrestoBlue Cell Viability Reagent, LIVE/DEAD Viability/Cytotoxicity Kit (for mammalian cells), Actin-Red 555 ReadyProbes Reagent (rhodamine phalloidin), and 4′,6-diamidino-2-phenylindole dihydrochloride (DAPI) were supplied by Invitrogen (Life Technologies, Thermo Fisher Scientific, Waltham, Massachusetts, USA). TritonX-100 (2-[4-(2,4,4-trimethylpentan-2-yl)­phenoxy]­ethan-1-ol), paraformaldehyde (PFA, HO­(CH_2_O)_n_H), glutaraldehyde solution (grade II, 25% in H_2_O, OHC­(CH_2_)_3_CHO), sodium cacodylate trihydrate ((CH_3_)_2_AsO_2_Na·3H_2_O, ≥98.0%, and Trypan Blue Powder (BioReagent, suitable for cell culture) were supplied by Sigma-Aldrich (St. Louis, MO, USA). Trypsin-EDTA 10× was supplied by Euroclone (AddLife AB, Stockholm, Sweden).

Leeches (Hirudo verbana, Annelida, and Hirudinea) were kindly donated by Italian Leech Farm (ILFARM SRL, Italy).

### Mineralized GelMgHA Hybrid Particles Development

A hybrid powder made of MgHA nanocrystals grown on Gel matrix was obtained through a biomineralization process already described by Campodoni et al.^34^ In detail, the heterogeneous nucleation of 80% of MgHA nanocrystals on assembling Gel matrix was achieved by means of a neutralization reaction performed as follows: an aqueous acid solution was prepared by mixing H_3_PO_4_ (2.31 g in 100 mL) in Gel aqueous solution (0.84 g in 33.5 mL) at room temperature; meanwhile, a basic solution was prepared by adding 0.34 g of MgCl_2_ to aqueous suspension of Ca­(OH)_2_ (2.60 g in 167 mL) kept at room temperature. The acid solution was immediately dropped into the basic solution under constant hand stirring at room temperature. The precipitated GelMgHA hybrid composite was matured in the mother liquor without stirring for 2 h, then was collected by centrifugation, washed three times with distilled water, and freeze-dried (Figure 1). As a last step, a micronizer was used to reduce the particles to a micrometric size and facilitate their dispersion within the polymeric hydrogel formulation.

### Hybrid Single-Layer Patches Development

Three different hybrid, single-layer patches with varying compositions and morphologies were developed. To set up the best polymer composition, two different hydrogels were prepared: specifically, a blend of gelatin and chitosan with a 3:1 ratio (GelMgHA@GelChit3:1) and a blend of chitosan and glycerol, with the role of improving the plasticity and flexibility of the material, in a 2:1 ratio to each other (GelMgHA@ChitGly2:1). For both compositions was added an amount of GelMgHA particles to obtain a total content of mineral phase (MgHA) of 30% with respect to the polymer. To study the contribution of different porosities, the blend with chitosan and glycerol was cast using two different fabrication methods: freeze-drying, generating a highly porous structure, and solvent casting, generating a thin and more compact layer (Table 5).

**Table 5:** Patches Code and Chemical Composition

Code	Solution composition	Fabrication method
GelMgHA@GelChit3:1_freeze	Gelatin (2.2%w/w)	Freeze-drying
	Chitosan (0.7%w/w)	
	GelMgHA (1.8%w/w)	
GelMgHA@ChitGly2:1_freeze	Chitosan (2%w/w)	Freeze-drying
	Glycerol (1%w/w)	
	GelMgHA (1.8%w/w)	
GelMgHA@ChitGly2:1_casting	Chitosan (2%w/w)	Solvent casting
	Glycerol (1%w/w)	
	GelMgHA (1.8%w/w)	

#### GelMgHA@GelChit3:1 Patches Development

The preparation of GelMgHA@GelChit3:1_freeze patches was carried out as described by Campodoni et al.^34^ Briefly, 10 mL of a 12 wt % Gel aqueous solution was prepared by dissolving the polymer in water at 40 °C; then, 20 mL of Chit solution (prepared by dissolving 0.4 g of chitosan in a 1% acetic acid water solution to achieve a 2 wt % concentration) was added, and the resulting blend underwent mechanical stirring at 37 °C for 30 min. Meanwhile, an aqueous suspension containing GelMgHA hybrid particles (0.97 g in 25 mL) was prepared and treated for 10 min with a tip sonicator ultrasonic processor (VCX130, Sonics and Materials, United States) in an ice bath to disperse and break up GelMgHA flakes. The Gel–Chit blend was then introduced into the GelMgHA suspension at 37 °C and left under magnetic stirring overnight for thorough homogenization, resulting in the final hybrid composite GelMgHA@Gel-Chit3:1. Finally, the patches were obtained through freeze-drying, through a cycle performed with a controlled freezing ramp of −50 °C/h until −40 °C, followed by controlled heating of 5 °C/h from −40 °C to −5 °C, and 3 °C/h until 20 °C, lasting approximately 3 days under vacuum (*p* = 0.086 mbar). As a final step, the GelMgHA@GelChit3:1_freeze patch underwent a DHT treatment at 120 °C for 48 h under a pressure of 0.01 mbar to enhance scaffold stability through the formation of covalent bonds within the polymeric components (Figure 16a).

**Figure 16:**
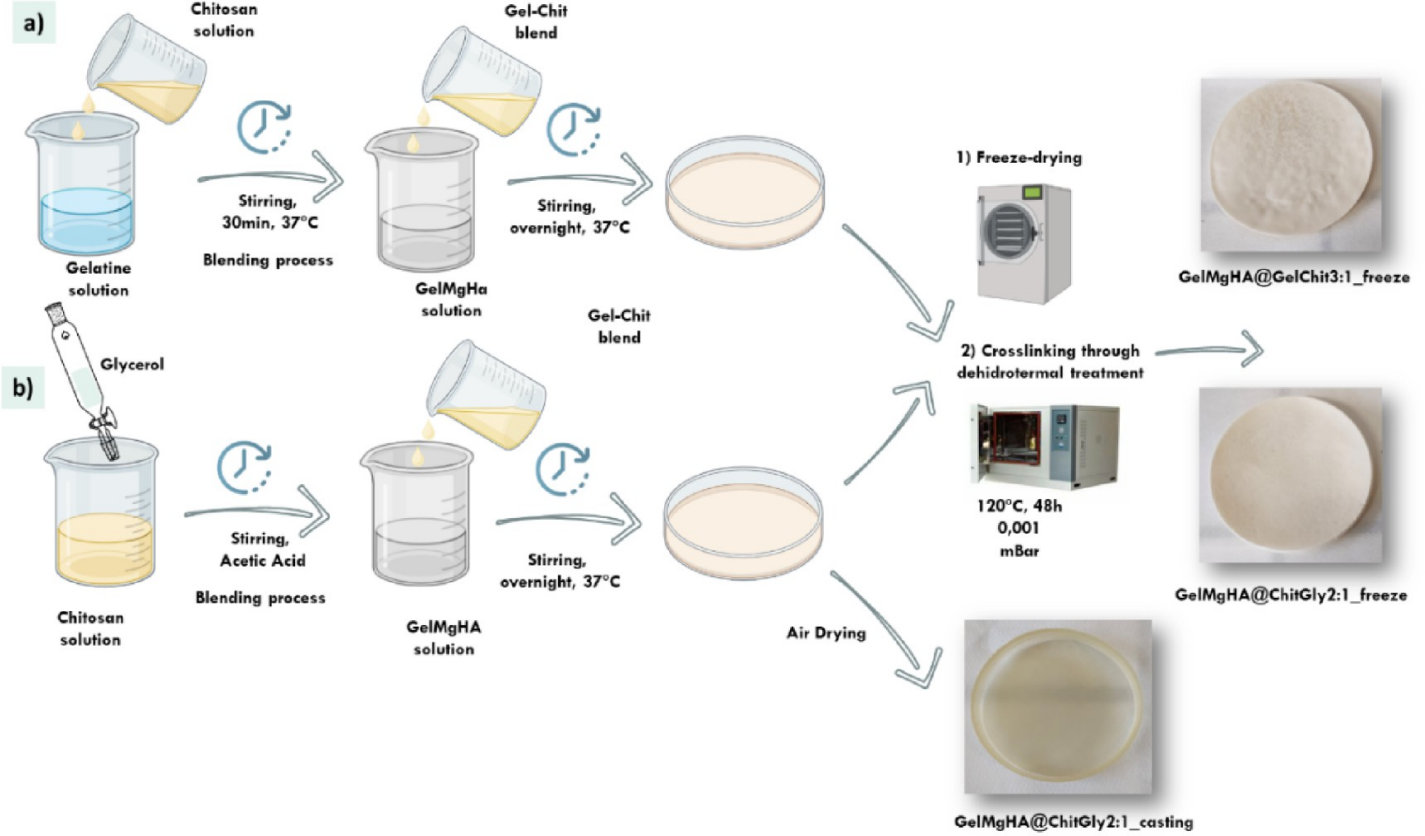
Schematic representation of the process for the preparation of hybrid single-layer patches. a) GelMgHA@GelChit3:1_freeze and b) GelMgHA@ChitGly2:1_freeze and GelMgHA@ChitGly2:1_casting.

#### GelMgHA@ChitGly2:1 Patches Development

Chitosan solution (3% w/w) was prepared by adding 2.4 g of chitosan powder in 80 g of acetic acid aqueous solution (1%, v/v) containing 1.2 g of glycerol as a plasticizer. An aqueous suspension containing GelMgHA hybrid particles (1.46 g in 40 mL) was prepared and treated for 10 min with tip sonicator ultrasonic processor (VCX130, Sonics and Materials, United States) in an ice bath to disperse and break up GelMgHA clusters. The Chit–Gly blend was then introduced into the GelMgHA suspension at 37 °C and left under magnetic stirring overnight for thorough homogenization, resulting in the final composite hydrogel GelMgHA@ChitGly2:1. Then, the patch GelMgHA@ChitGly2:1_freeze was obtained through freeze-drying: 20 g of the composite hydrogel was cast into a Petri dish (Ø 9 cm); then a freeze-drying cycle was performed with a controlled freezing ramp of −50 °C/h until −40 °C, followed by controlled heating of 5 °C/h from −40 °C to −5 °C, and 3 °C/h until 20 °C, lasting approximately 3 days under vacuum (*p* = 0.086 mbar). Lastly, to enhance the stability through a covalent bond formation, the GelMgHA@ChitGly2:1_freeze patch underwent a DHT treatment at 120 °C for 48 h under a pressure of 0.01.

Instead, the second type of patches GelMgHA@ChitGly2:1_casting were obtained through the solvent casting technique: 60 g of composite hydrogel was poured into a Petri dish (Ø 9 cm) and let dry at room temperature in a fume hood for 48 h until complete solvent evaporation (Figure 16b).

#### Bilayer Patches Development

For the preparation of the bilayered patches, GelMgHA@ChitGly2:1 hydrogel, prepared as described in GelMgHA@ChitGly2:1 Patches Development section, was poured into a Petri dish covered at the bottom with a Mylar film (for later easier detachment), and let dry in a fume hood for 24 h, then in an oven at 40 °C for about 3–4 h, until a firm but still adhesive surface was obtained.

The partially dried film was removed from the Petri dish and placed on top of the freeze-dried porous bottom layer (alternatively GelMgHA@GelChit3:1_freeze and GelMgHA@ChitGly2:1_freeze, see Table 6) obtained as described in GelMgHA@GelChit3:1 Patches Development and GelMgHA@ChitGly2:1 Patches Development sections. Then, the Mylar film was removed, and the top layer was let dry completely under a fume hood for an additional 24 h (Figure 17), until the obtainment of two bilayer patches with compositions as reported in Table 6.

**Figure 17:**
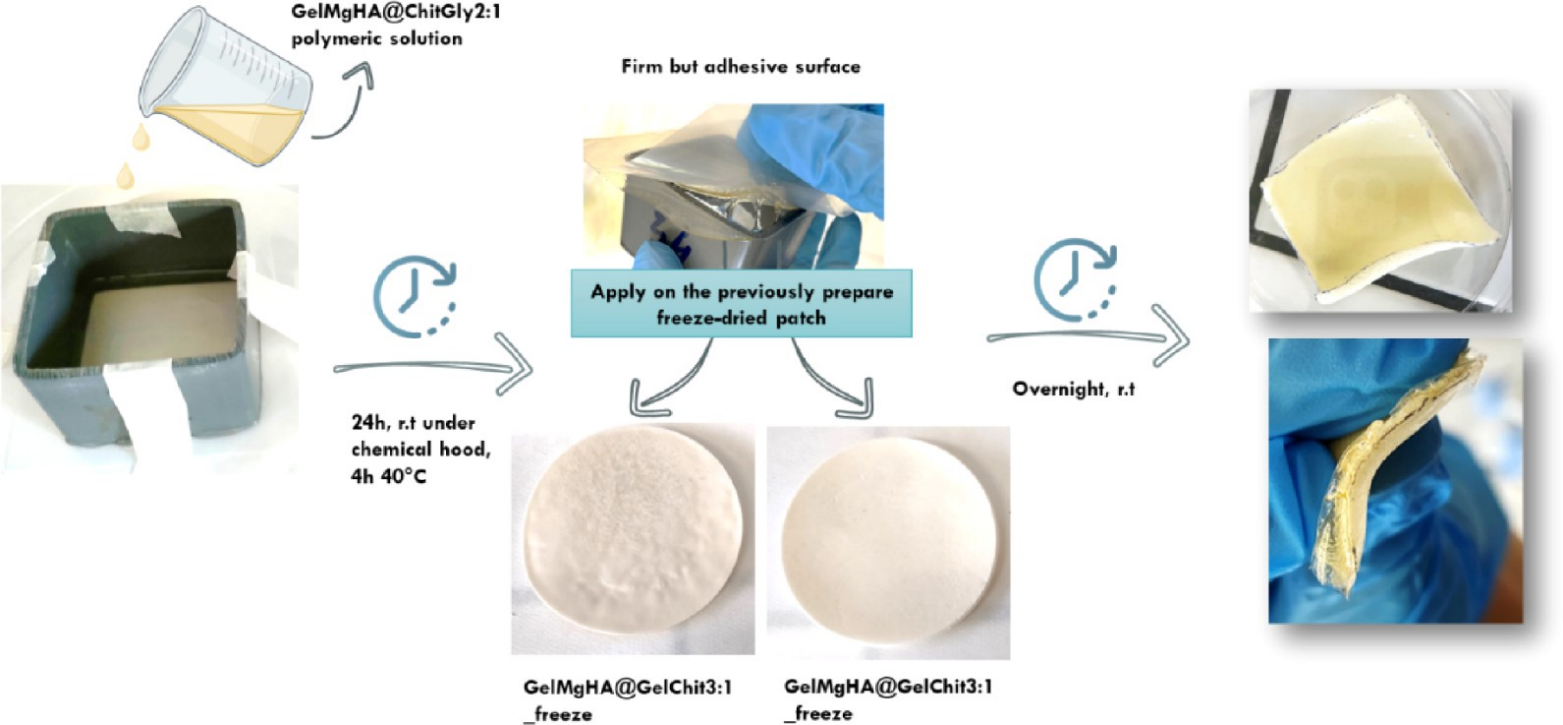
Schematic representation of bilayer patches preparation.

**Table 6:** Bi-Layers Code and Chemical Composition

Code	Bottom-layer	Top-layer
BL-1	GelMgHA@GelChit3:1_freeze	GelMgHA@ChitGly2:1_casting
BL-2	GelMgHA@ChitGly2:1_freeze	GelMgHA@ChitGly2:1_casting

### Characterization

#### Morphological Characterization

Powder morphology was examined by scanning electron microscopy (Field Emission Gun Scanning Electron Microscope, FEI, Quanta 200, USA—FEG-SEM). The specimens were previously mounted on aluminum stubs by means of carbon tape and platinum/palladium coated using a coating unit Polaron Sputter Coater E5100 (Polaron Equipment, Watford, Hertfordshire, UK). The patch morphology and the pore size were observed by environmental scanning electron microscopy (SEM TM Quanta 200, FEI, Thermo Fisher Scientific Inc.), set in high vacuum (*p* < 10^–4^ Torr) mode. The samples were fixed on aluminum stubs using carbon tape and they were coated with Au using coating units Polaron Sputter Coater E5100 (Polaron Equipment, Watford, Hertfordshire, United Kingdom).

Patch macroporosity assessment was performed by using the water-squeezing method. It quantifies water content within a scaffold before and after compression, operating on the premise that water resides in both polymer bounds and small and large pores, with macropores crucial for cell infiltration and growth. To determine the macropore volume percentage, the scaffold was immersed in deionized water for 1 h and weighed (*M*_swollen_). It was then compressed to expel pore water and reweighed (*M*_squeezed_). The macropore volume percentage was determined using the following equation:

1 $$\mathrm{Macropore volume percentage}\left(\%\right)=(\left(M_{\mathrm{swollen}}-M_{\mathrm{squeezed}}\right)/M_{\mathrm{swollen}})\times 100$$

The values were expressed as the mean ± standard error (*n* = 3).^34^

#### Chemical Characterization

##### Inductive Coupled Plasma Optical Emission Spectroscopy (ICP-OES)

Inductively Coupled Plasma-Optical Emission Spectrometry (ICP-OES, Agilent Technologies 5100, Santa Clara, USA) was conducted using a Liberty 200 spectrometer (Varian, Palo Alto, United States) for the quantitative determination of Mg^2+^, Ca^2+^, and PO_4_^3–^ ions, which constitute the inorganic mineral component. In brief, 10 mg of hybrid GelMgHA particles or 20 mg of hybrid patch samples were dissolved in 50 mL of a 2 wt % HNO_3_ solution prior to the analysis.

#### X-ray Diffraction (XRD)

XRD patterns were obtained by using a D8 Advance diffractometer (Bruker, Karlsruhe, Germany) equipped with a LynxEye position-sensitive detector. The analysis employed Cu Kα radiation (λ = 1.54178 Å) at 40 kV and 40 mA. Spectra were recorded in the 2θ range from 20° to 80 °C, with a step size (2θ) of 0.02° and a counting time of 0.5 s.

#### Thermogravimetric Analysis (TGA)

The thermal properties of the samples were assessed using an STA 449 F3 Jupiter instrument (Netzsch, Gerätebau, Germany) to confirm either the correct nucleation of 80% of MgHA nanocrystals on the gelatin matrix or the correct incorporation of 30% of the mineral phase (relative to the polymer content) in the patches. Simultaneous thermogravimetric analysis (TGA) and differential scanning calorimetry (DSC) were conducted in alumina crucibles, from room temperature to 1100 °C, at a heating rate of 10 °C/min under a nitrogen flow. The sample weighed approximately 10 mg.

#### Physical Characterization

##### Swelling Behavior

To assess the fluid uptake capacity, cylindrical samples of the patch (diameter: 6 mm, height: 0.2–2 mm) were immersed in a PBS solution at 37 °C until saturation was reached. At specified intervals (0.5, 1, 2, 4, 6, 24, and 48 h), samples were removed, excess water was removed using filter paper, and then weighed. The equilibrium swelling ratio was determined using the formula:

2 Swelling ratio=(wet weight−dry weight)/(wet weight)×100

The measure was repeated in triplicate on different samples.^68^

#### In Vitro Degradation

For degradation assessments, cylindrical patch samples (diameter: 6 mm, height: 0.2–2 mm) were placed in phosphate-buffered saline (PBS, pH 7.2) with 0.1% (wt/vol) NaN_3_ at 37 °C. In the case of patches obtained through the solvent casting technique, an initial freeze-drying step was performed to eliminate any residual water from the samples. At specific time intervals (1, 3, 7, and 14 days), samples were retrieved, washed twice with Milli-Q water, freeze-dried for 2 days, and then reweighed. The degradation percentage (*D* (%)) was calculated using the equation:

3 $$D\left(\%\right)=\left(W_{i}-W_{f}\right)/W_{i}\times 100$$

where *W_i_* is the initial weight of the freeze-dried sample before immersion in PBS, and *W_f_* is the weight of the freeze-dried sample at a specific time point.^68^

#### Water Vapor Transmission Rate (WVTR)

Moisture permeability of the prepared patch was determined following the ASTM E96 standard method.^69^ Initially, a vial (diameter of 1.50 cm) containing 10 mL of Milli-Q water was covered with the patch as a lid and weighed (*W*0). An open vial and a vial sealed with its lid served as positive and negative controls, respectively. After placement in a humidity chamber at 37 °C and 50% relative humidity for 24, 48, and 72 h, they were reweighed (*W*t). The WVTR of the patch was computed with the formula:

4 $$\mathrm{WVTR}\left({\mathrm{g m}}^{\left(-2\right)}d^{\left(-1\right)}\right)=\left(\left(W_{t}-W_{0}\right)/t\right)/A$$

where *A* is the exposed area of the patch (m^–2^), and *t* is the time of measurement (days). The values were expressed as the mean ± standard error (*n* = 3).

### Medication Patches with Vancomycin and Release Evaluation

For the preparation of medicated samples, samples were cut in cylinders (diameter: 6 mm, height: 0.2–2 mm) and preswelled in a phosphate buffer saline (PBS, pH 7.2) at 37 °C for 30 min. A solution of VNC was prepared by dissolving the antibiotic in PBS to achieve a concentration of 50 mg/mL. Following the swelling period, the samples were removed from the buffer, excess liquid was gently removed with filter paper, and 30 μL of the antibiotic solution was soaked in each sample, resulting in a loading of 1.5 mg of VNC per sample. This dosage was determined considering the experimental setup’s feasibility, including the diagnostic technique’s detection limit, as well as the minimum inhibitory concentration (MIC) of the chosen antibiotic. To ensure uniform drug distribution within the patches, the samples were subsequently agitated in a shaker for approximately 20 min.

The release profile of VNC from the patches was evaluated by incubating the loaded samples in 5 mL of PBS (pH 7.4) in a thermostatic incubator shaker at 37 °C, mimicking dynamic in vivo conditions. At specified intervals (30 min, 1, 3, 5, 7, 24, 48, and 72 h), 10% (500 μL) of the release solution was collected and replaced with an equivalent volume of fresh PBS to maintain the uniformity of the release solution. The cumulative concentration of released VNC was determined using a UV–vis-NIR spectrophotometer Lambda 750 (PerkinElmer Instrument, USA) at 280 nm. Nonmedicated scaffolds served as the reference. Quantification limits for VNC were established, and the released antibiotic quantity was calculated using a calibration curve recorded with standard VNC solutions.

### Antimicrobial Evaluation

#### In Vitro Assessment of Patches’ Antibacterial Activity

The antibacterial properties of the patches were evaluated in vitro against two reference Gram-positive bacteria, Staphylococcus aureus (ATCC 25923) and Staphylococcus epidermidis (ATCC 12228). Laboratory strains were obtained from the American Type Culture Collection (ATCC), routinely grown on 5% blood agar plate (Biolife Italiana S.r.l., Milan, Italy), and freshly used in the antibacterial assays.

The inhibitory activity of the samples was assayed by measuring the diameters of the bacterial-free zone obtained in a standardized disk diffusion assay, as previously described.^70^ For the experiment, bacterial suspensions were prepared in PBS solution and adjusted to an approximate optical density (at 630 nm) of 0.08–0.1. The working solution was inoculated on the surface of the Mueller–Hinton agar plate (MHA) (Sigma-Aldrich, St. Louis, MO, USA), and the VNC-loaded patches were placed on the agar surface. As a control, a sterile paper disk was previously loaded following the same procedure for the Vancomycin patches and included in the assay. After 24 h of incubation at 37 °C, the agar plates were observed, and the diameters of the inhibition zone were measured to the nearest whole millimeter with a ruler. All experiments were performed in duplicate on different days.

In addition, the antibacterial activity of the patches was assessed by testing the inhibitory effect of VNC released by the loaded samples in a liquid solution at different time intervals. For this purpose, VNC-loaded patches were incubated in 500 μL of PBS at 37 °C. At each time point (30 min, 1 h, 3 h, 5 h, 7 h, 24 h, 48 h, 72 h), the solution was collected and replaced with 500 μL of fresh PBS. The liquid samples were assayed by using a well-established broth microdilution method,^46^ in compliance with the international guidance documents (CLSI, EUCAST). Briefly, bacterial suspensions, prepared as previously described, were diluted 1:200 in Mueller–Hinton Broth (MH) (Biolife Italiana S.r.l., Milan, Italy) and then incubated with a 10-fold dilution of the starting liquid samples and subsequent serial 2-fold dilutions. Bacterial growth was spectrophotometrically measured at 630 nm after 24 h of incubation at 37 °C. Measurements were performed in triplicate on three independent biological replicates.

Loaded patches, harvested at the end of this experiment (72 h of incubation in PBS), were tested for their residual inhibitory potential on S. aureus and S. epidermidis by means of the disk diffusion assay, as previously described.

#### Sample Preparation for SEM Analysis

S. aureus and S. epidermidis were cultured overnight at 37 °C in MH broth and then 1:10 diluted in the same culture medium. Aliquots of these suspensions (500 μL) were transferred into a 24-well plate together with the bilayered patches, loaded with VNC and unloaded as control. After 6 h of incubation at 37 °C, samples were harvested, washed twice with cold cacodylate 0.1 M, and fixed with 2.5% (v/v) glutaraldehyde in cacodylate at 4 °C for 2 h. Finally, patches were washed in 0.1 M sodium cacodylate buffer (pH 7.4), followed by freeze-drying for 2 days. Samples were mounted on aluminum stubs and gold-sputtered by a Polaron Sputter Coater E5100 (Polaron Equipment, Watford, Hertfordshire, UK). Imaging was performed using Stereoscan 360 SEM (Cambridge Instruments, UK) under high vacuum conditions.

### In Vitro Cytocompatibility Assay

#### Cell Culture

WS1 human skin fibroblast cells (ATCC CRL-1502) were purchased from the American Type Culture Collection (ATCC). The cells were cultured in complete cell culture medium consisting of α-MEM supplemented with 10% fetal bovine serum (FBS) and 1% penicillin-streptomycin (P/S). Cells were maintained in 75 cm^2^ culture flasks at 37 °C in a humidified atmosphere with 5% CO_2_. Once the cells reached approximately 80% confluency, they were detached using 3 mL of 10× trypsin in PBS (1:9) for 3 min and subsequently centrifuged at 1000 rpm for 5 min. The resulting cell pellet was resuspended in complete culture medium to achieve a final cell density of 15 000 cells per 10 μL for seeding preparation. To seed the scaffolds, 15 000 cells in 10 μL of medium were slowly applied dropwise onto each scaffold. The seeded scaffolds were incubated at 37 °C with 5% CO_2_ and controlled humidity. The culture medium was replaced every 2–3 days.

#### Scaffold Preconditioning and Drug Loading

For the in vitro experiment, two different procedures were carried out: (i) testing the single-patch on its own (named as reported in Table 1) and (ii) after drug loading and drug release. Briefly, the patches suitable for drug loading (GelMgHA@GelChit3:1_freeze and GelMgHA@ChitGly2:1_freeze, see Table 3) were loaded with VNC following the protocol reported in Medication Patches with Vancomycin and Release Evaluation section, and then evaluated after 24 h of release in cell culture medium at 37 °C during the preconditioning step (patches named U-GelMgHA@GelChit3:1_freeze and U-GelMgHA@ChitGly2:1_freeze), to study the patch cytocompatibility and its ability to preserve it even after drug loading and release.

Before cell seeding, both patches (on their own and loaded) were preconditioned in a complete cell culture medium for 24 h to ensure protocol consistency. Each scaffold was placed into separate wells of a 48-well plate, and 800 μL of complete culture medium was added to each well.

#### Cell Viability Assay

Cell viability was assessed at days 1, 3, and 7 postseeding using the PrestoBlue Cell Viability Reagent, following the manufacturer’s instructions. Briefly, scaffolds were incubated with a 10% (v/v) PrestoBlue solution for 2 h at 37 °C in a humidified 5% CO_2_ atmosphere. Each scaffold and solution were transferred to a separate 2 mL Eppendorf tube and mechanically crushed using a mortar. The mixture was centrifuged at 6000 rpm for 1 min to precipitate debris. The supernatant was then analyzed using a Fluoroskan Microplate Fluorometer (Thermo Fisher Scientific, Waltham, Massachusetts, USA) with excitation and emission wavelengths of 544 and 590 nm, respectively. A biological triplicate with technical triplicate was analyzed for each time point.

#### Live and Dead Assay

A LIVE/DEAD assay was conducted on days 1, 3, and 7 postseeding to qualitatively assess the viability of cells within the various scaffolds, following the manufacturer’s instructions. Briefly, the scaffolds were rinsed with 1× PBS and incubated for 15 min in a solution containing 2 μM Acetoxymethyl Calcein (AM-calcein) and 4 μM Ethidium Homodimer-1 (EthD-1) in 1× PBS at 37 °C under a humidified atmosphere with 5% CO_2_. After incubation, the scaffolds were washed with 1× PBS and imaged using a Nikon Ti-E inverted fluorescence microscope (Japan) using FITC and TRITC filters to visualize live and dead cells, respectively.

#### Cell Morphology Analysis

Qualitative evaluation of cell morphology was conducted via Actin and DAPI staining at days 1, 3, and 7 postseeding. Both cell-seeded patches and 2D cell cultures were analyzed. Samples were first fixed with 4% paraformaldehyde (PFA) for 15 min, followed by permeabilization with 0.1% (v/v) Triton X-100 in 1× phosphate-buffered saline (PBS) for 5 min. Samples were then incubated in the dark with ActinRed 555 ReadyProbes Reagent for 30 min, followed by DAPI (600 nM) for 7 min. Between each step, samples were washed with 1× PBS. Imaging was performed using an inverted Ti-E fluorescence microscope (Nikon, Japan).

#### Scanning Electron Microscopy (SEM) Analysis

To assess the morphology of the cells following culture on the patches, SEM analysis was performed on cell-seeded patches on day 3, using one biological replicate per sample. Samples were prepared with the same procedure already described in the second paragraph of Sample Preparation for SEM Analysis section.

### In Vivo Studies on Medicinal Leeches

#### Animals and Treatments

Animals were maintained and used in accordance with the regulations on animal experimentation at the University of Insubria. Leeches (Hirudo verbana) measuring 10 cm were kept in tap water at 20 °C in aerated tanks. Leeches were used as hosts for the following patches: single-layer GelMgHA@ChitGly2:1 and GelMgHA@GelChit3:1, bilayer BL-1 (top layer: GelMgHA@ChitGly2:1; bottom layer: GelMgHA@GelChit3:1), and bilayer BL-2 (top layer: GelMgHA@ChitGly2:1; bottom layer: GelMgHA@ChitGly2:1). Three animals for each patch and for each time point (72 h and 7 days) were used. Before each experiment, leeches were anesthetized with a 10% ethanol solution and then dissected.

For the patch grafting, at about the 80th superficial metamere from the oral sucker, a block of 2 mm × 2 mm × 2 mm was excised from the leech body wall. Afterward, a piece of scaffold of 2 mm × 2 mm × 2 mm was placed in the same hollow. Grafts were sutured with Dafilon surgical synthetic monofilament (B. Braun) to avoid patch loss due to contraction of the muscular body wall. Grafted leeches were kept in moist chambers for a postsurgical recovery period of 2 h and subsequently placed in water tanks. The rate of successful transplantation experiments for GelMgHA@ChitGly2:1_freeze, BL-1, and BL-2 graft types was 80%, while for GelMgHA@GelChit3:1_freeze, it was of 10%. The leeches that survived the surgical operation were able to move following recovery from anesthesia. The leech body tissues containing the samples were removed at specific time points after the treatments.

#### Optical Microscopy and Indirect Immunofluorescence Staining

Leech tissues, dissected from the area containing the grafted patches, were embedded in Polyfreeze tissue freezing medium (Polysciences, Eppelheim, Germany) and immediately frozen in liquid nitrogen. Cryosections (7 μm in thickness) were obtained with a Leica CM1850 cryotome, and slides were immediately used or stored at −20 °C. Cryosections were rehydrated with PBS for 5 min and stained by crystal violet and basic fuchsine for a morphological view. For indirect immunofluorescence, samples were washed with PBS and then preincubated for 30 min with PBS containing 2% bovine serum albumin (BSA) before the primary antibody incubation (1 h at room temperature, RT). The primary antibody used was the polyclonal rabbit antihuman CD154 (Proteintech, Germany), which reacts with leech myofibroblast cells^62^ diluted 1:100. The washed specimens were incubated for 1 h at RT with the secondary antibody, goat antirabbit Cy3-conjugated (excitation 562 nm, emission 576 nm), diluted 1:200 (Jackson ImmunoResearch Laboratories, West Grove, PA, USA). In the control samples, primary antibodies were omitted, and sections were treated with BSA-containing PBS.

Nuclei of the cells were stained with DAPI (4’,6-diamidino-2-phenylinedole, 0.1 mg/mL diluted in PBS) for 3 min. Slides were mounted with Cityfluor (Cityfluor Ltd., UK) coverslips and observed under a fluorescence microscope (Nikon Digital Sight DS-SM, Tokyo, Japan). Images were captured with a DS-5M-L1 digital camera (Nikon). Images were combined with Adobe Photoshop (Adobe Systems, San Jose, CA, USA).

## Supplementary Material



## Notes

Disk diffusion assays, antibacterial activity against S. epidermidis and SEM images, and A and D tests (PDF)
