# Supplementary material for: From Wound Dressing to Tissue Regeneration: Bilayer Medicated Patches for Personalized Treatments of Chronic Wounds
Source: ACS Appl Mater Interfaces. 2025 Jun 5;17(24):35240–61. doi: 10.1021/acsami.5c06444 (PMC12186226; doi:10.1021/acsami.5c06444)
Supplement: Supplementary file 1 [file am5c06444_si_001.pdf]

SUPPORTING INFORMATION

From wound dressing to tissue regeneration: bi-layer medicated patches for personalized treatments of chronic wounds

Sara Bernardoni[1], Elisabetta Campodoni[1], Gaia Vicinelli[1], Mohamed Saqawa [1,2], Francesca Bonvicini[3], Laura Pulze[4], Nicolò Baranzini[4], Giorgia Costantini[4], Monica Montesi[1], Giovanna Angela Gentilomi[3-5], Annalisa Grimaldi[4], Monica Sandri[1*]*

[1] Institute of Science Technology and Sustainability for Ceramics (ISSMC), National Research Council (CNR), Via Granarolo 64, 48018 Faenza, Italy

[2] Department of Chemical, Biological, Pharmaceutical and Environmental Sciences, University of Studies of Messina, 98122 Messina (ME), Italy

[3] Department of Pharmacy and Biotechnology, Alma Mater Studiorum - University of Bologna, Via Massarenti 9, 40138 Bologna, Italy;

[4] Department of Biotechnology and Life Science, University of Insubria, Via Dunant 3, 21100 Varese, Italy

[5] Microbiology Unit, IRCCS Azienda Ospedaliero-Universitaria di Bologna, Via Massarenti 9, 40138 Bologna

Corresponding authors: elisabetta.campodoni@issmc.cnr.it; monica.sandri@issmc.cnr.it

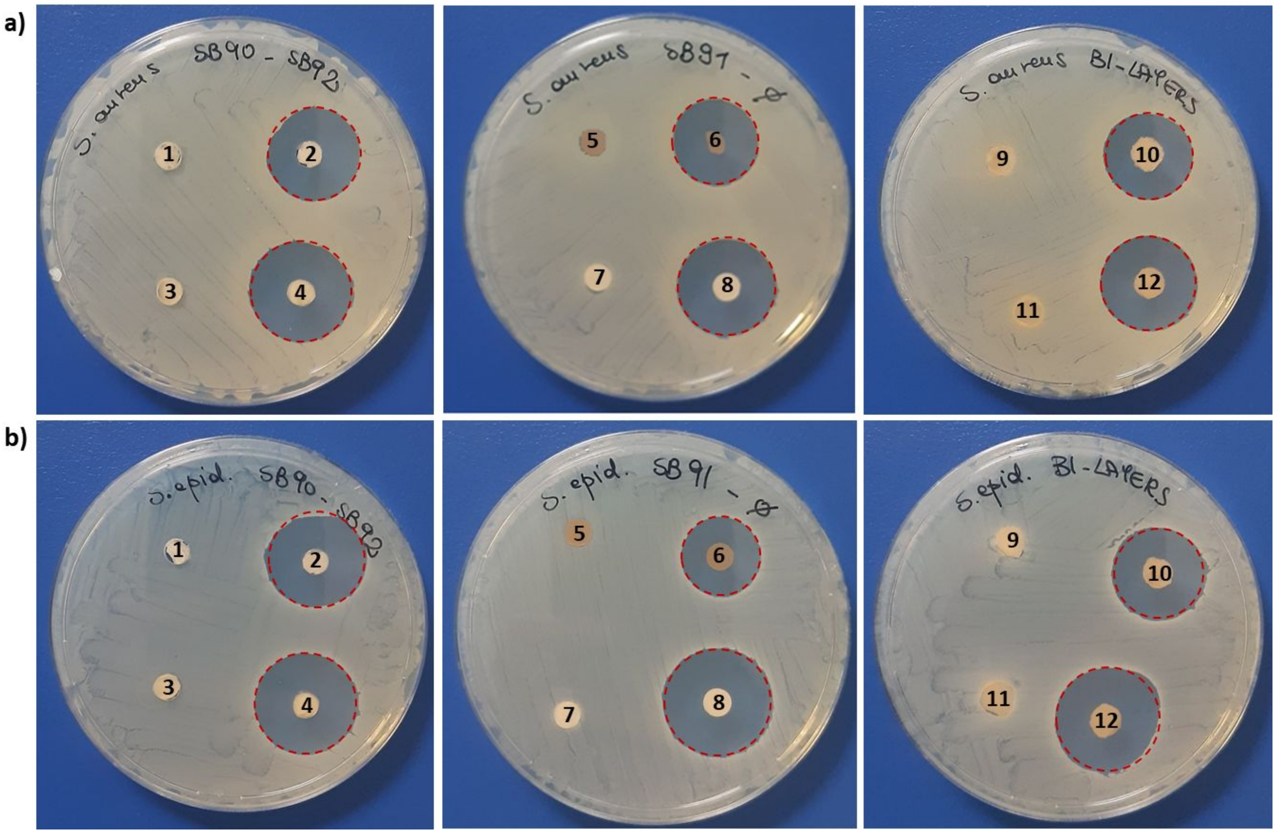

Figure S1: Disk diffusion assays on a) *S. aureus* and b) *S. epidermidis*. Plates were spread with the bacterial suspensions, thereafter the VNC loaded patches and unloaded, as controls, were placed on the agar surface. After an overnight incubation at 37°C, the diameter of the clear halo around the patches was measured using a ruler. List of tested samples: GelMgHA@GelChit3:1_freeze and the corresponding VNC loaded sample (1 and 2); GelMgHA@ChitGly2:1_casting and the corresponding VNC loaded sample (3 and 4); GelMgHA@ChitGly2:1_freeze and the corresponding VNC loaded sample (5 and 6); sterile paper disk and the VNC loaded disk (7 and 8); the BL-1 and the corresponding VNC loaded sample (9 and 10); the BL-2 and the corresponding VNC loaded sample (11 and 12).

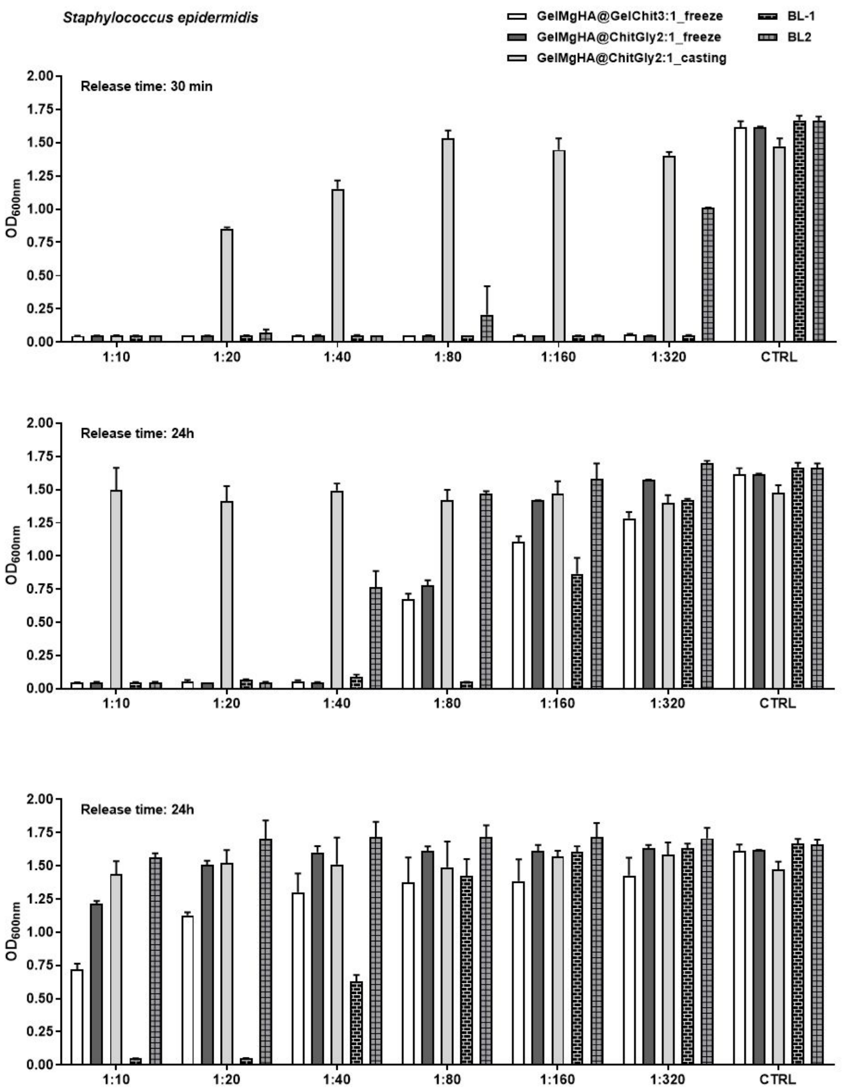

Figure S2: Antibacterial activity against *S. epidermidis* of the VNC released in PBS solutions, at different time intervals, from the patches. Data are the means ± SD of the Optical Density at 600nm. CTRL, positive growth controls.

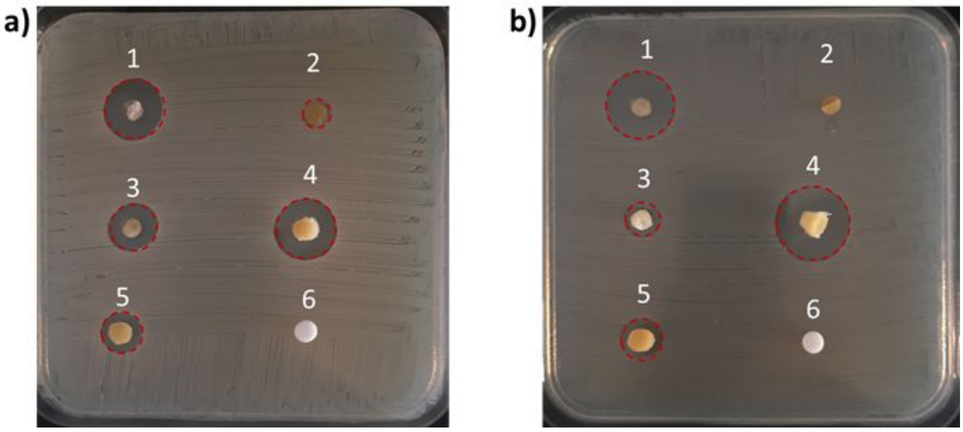

Figure S3: Disk diffusion assays on a) *S. aureus* and b) *S. epidermidis.* Plates were spread with the bacterial suspensions thereafter the VNC loaded patches after 3 days of incubation in PBS were collected and placed on the agar surface. After an overnight incubation at 37°C, the diameter of the clear halo was measured using a ruler. List of tested samples: GelMgHA@GelChit3:1_freeze (1); GelMgHA@ChitGly2:1_casting (2); GelMgHA@ChitGly2:1_freeze (3); BL-1 (4); BL-2 (5); sterile paper disk (6).

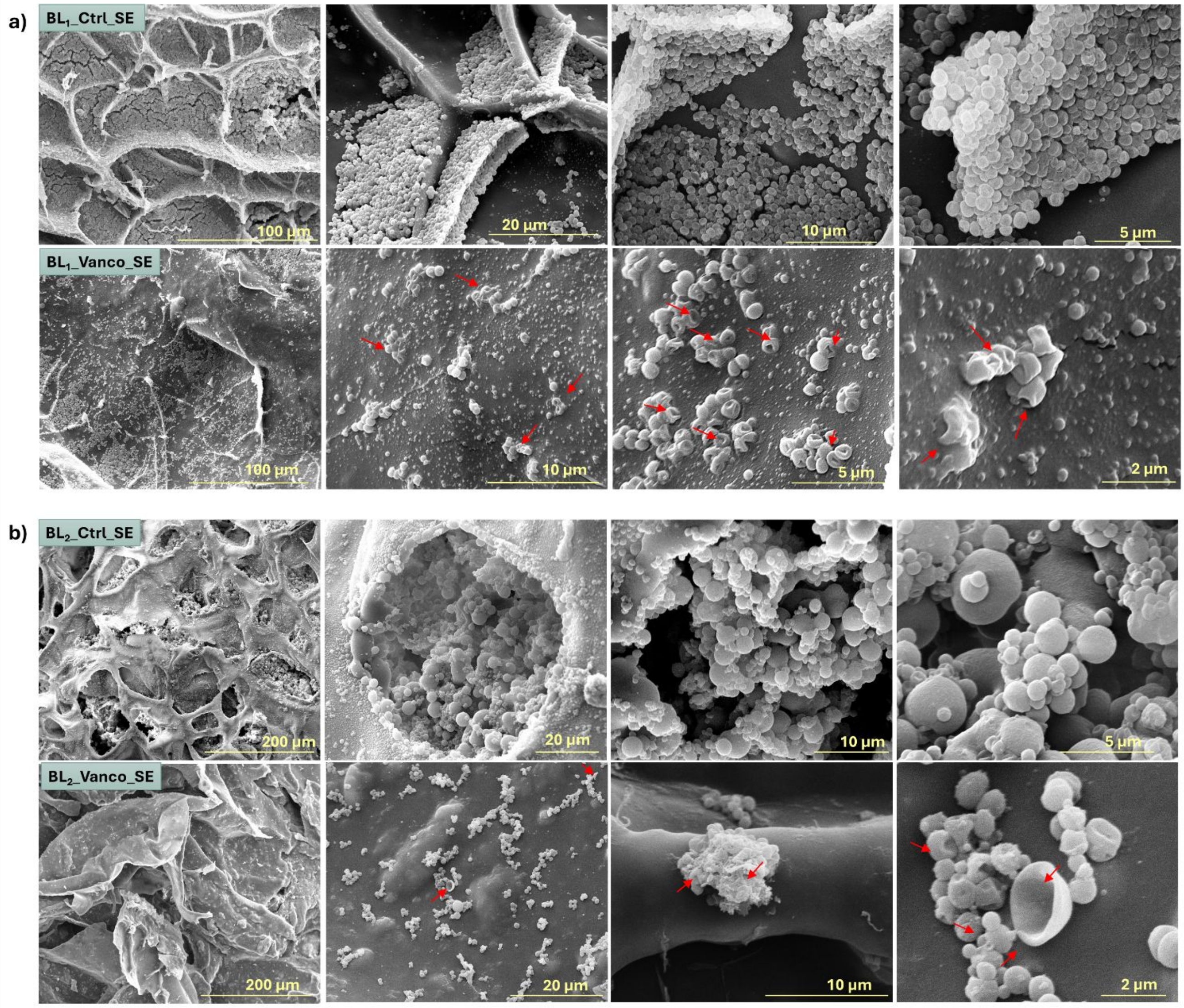

Figure S4: Scanning electron microscopy (SEM) analysis of BL-1 and BL-2 samples both unloaded and loaded with Vancomycin, after 6 hours of incubation with S. epidermidis (SE). a) The images refer to BL-1, with unloaded samples at the top and samples loaded with Vancomycin at the bottom. b) The images refer to BL-2, with unloaded samples at the top and samples loaded with Vancomycin at the bottom. Red arrows indicate bacteria with evident alterations of the membrane in the Vancomycin treated patches

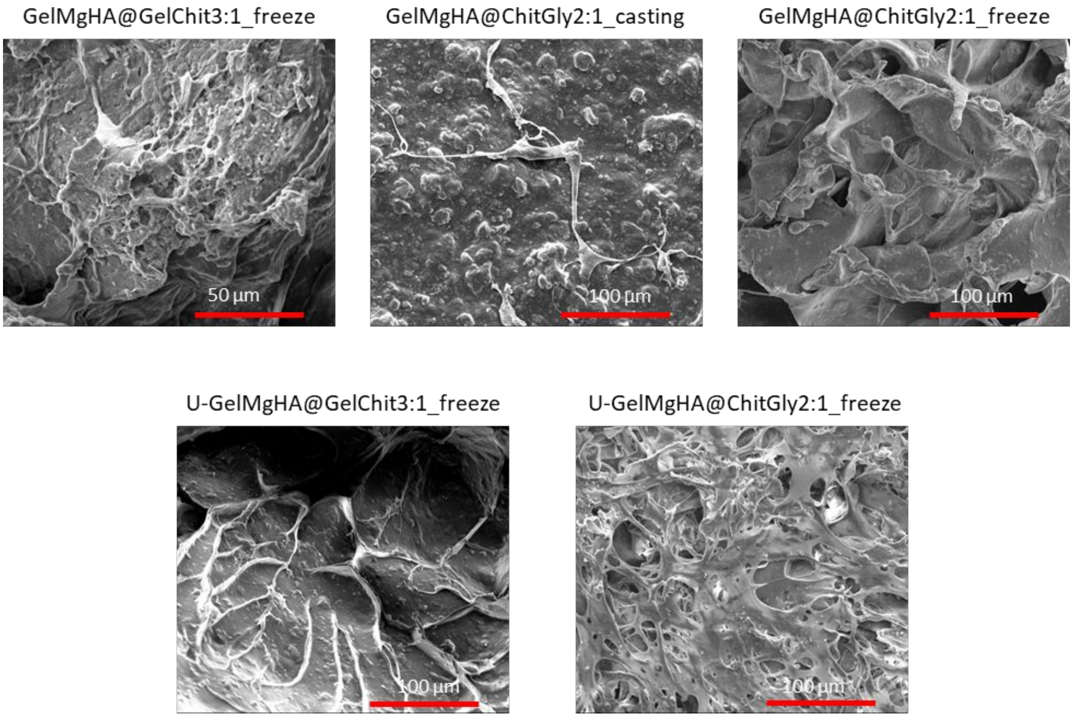

Figure S51: A representative images of SEM analysis of all the scaffolds tested after 3 days post-seeding.

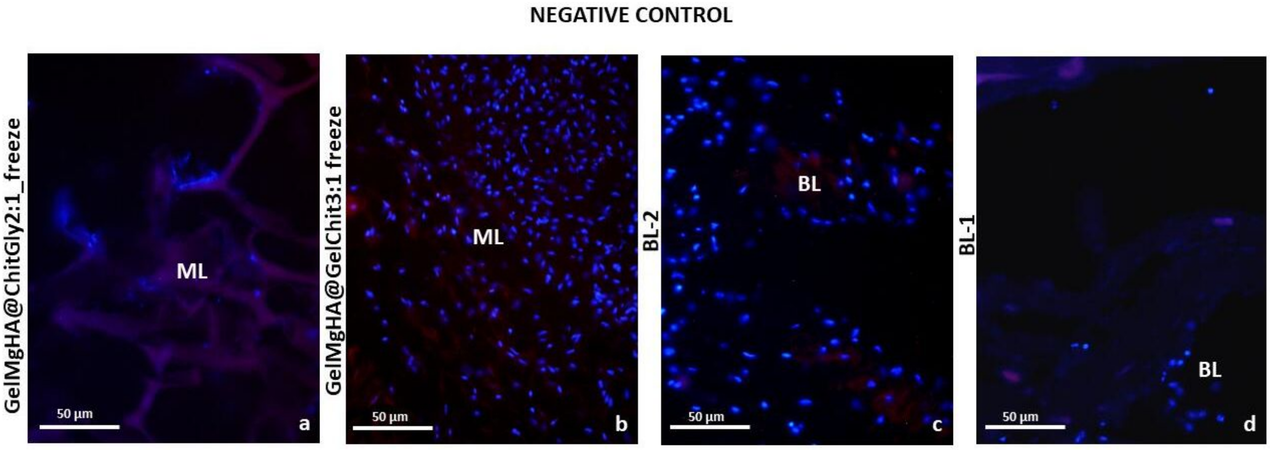

Figure S6: Cross-sections of a leech body wall grafted with the monolayers (ML) GelMgHA@ChitGly2:1_freeze (a), GelMgHA@GelChit3:1_freeze (b), and the bi-layers BL-2 (c) and BL-1 (d) after 7 days post-implantation. No CD154-positive cells are detectable. Nuclei are stained with DAPI (blue).
